# Activating GCN2 and subsequently the Unfolded Protein Response with the small oral molecule NXP800 delays tumor growth in osteosarcoma

**DOI:** 10.1038/s41420-026-02941-2

**Published:** 2026-02-05

**Authors:** Emma Racineau, Morgane Lallier, Anaïs Postec, Jérôme Amiaud, Rose-Anne Thépault, Régis Brion, Séverine Battaglia, Céline Charrier, Marie-Anne Colle, Bénédicte Brounais-Le Royer, Marc Baud’huin, Franck Verrecchia, Benjamin Ory, Steven Georges, François Lamoureux

**Affiliations:** 1 CRCI2NA, Inserm UMR 1307, CNRS UMR 6075, Université d’Angers, Nantes Université, Nantes, France; 2 CHU de Nantes, Nantes, France; 3 Oniris, INRAE, PAnTher, Nantes, France

**Keywords:** Targeted therapies, Paediatric cancer, Sarcoma

## Abstract

Osteosarcoma (OS) is the most common primary malignant bone tumor mainly affecting children and young adults. Despite current treatments combining polychemotherapy and surgery, survival rates have remained unchanged for decades, highlighting the need to identify novel therapeutic approaches. NXP800, a newly developed orally available molecule, represents a promising therapeutic option. The therapeutic efficacy of NXP800 was evaluated in vitro and in a preclinical murine xenograft model of OS. RNA-seq analysis and functional assays were conducted to investigate the mechanisms of action and molecular target of NXP800. NXP800 decreases the viability of OS cell lines by blocking proliferation and inducing apoptosis. Mechanistically, NXP800 activates the Unfolded Protein Response (UPR), as demonstrated by eIF2α phosphorylation and ATF4 upregulation. This effect is mediated through the engagement of the Integrated Stress Response (ISR) via the activation of GCN2 kinase. Inhibition of GCN2, either through molecular or pharmacological approaches, abolishes NXP800-induced eIF2α phosphorylation and partially restores OS cell viability. Furthermore, NXP800 activates the IRE1α/JNK/c-Jun pathway while increasing the expression of the pro-apoptotic protein Puma. Finally, NXP800 delays tumor growth in preclinical OS model by promoting apoptosis. This study is a preclinical proof-of-principle of therapeutic efficacy of NXP800 both in vitro and in vivo, highlighting the relevance of targeting GCN2, and consequently activating the ISR and UPR, to induce apoptosis and inhibit tumor progression in OS.

## Background

Osteosarcoma (OS) is the most common primary malignant bone tumor affecting children and young adults. Mainly localized in the extremities of long bones, OS is characterized by osteolytic lesions associated with ectopic bone formation and the development of pulmonary metastases, which worsen the prognosis [1]. Current treatment combines polychemotherapies (cisplatin, doxorubicin, methotrexate, and ifosfamide) associated with tumor resection [2]. This treatment results in a 5-year survival rate of 70% for localized forms, dropping drastically to 20–30% for bad responders to treatment, or for patients with pulmonary metastases at diagnosis [1]. There is an urgent need to develop new therapeutic strategies to improve outcomes for patients with OS.

A tumor cell is exposed to high levels of extrinsic stress, including hypoxia, pH alterations, or exposure to chemotherapies, but also intrinsic stress such as genetic and epigenetic modifications leading to the expression of oncogenes and repression of tumor suppressor genes [3]. In this context, tumor cells counteract the stress response mechanisms, including heat shock response (HSR) and unfolded protein response (UPR) as adaptative mechanisms to survive [4, 5]. Exploiting these stress adaptations as therapeutic vulnerabilities represents a promising strategy for cancer treatment.

Among them, the small molecule, NXP800, was initially develop to target the activity of the transcription factor Heat Shock Factor 1 (HSF1), which is the major regulator of the HSR [6, 7]. More recently, NXP800 has been described to activate the UPR in advanced treatment-resistant prostate cancer [7]. The UPR is a highly conserved cellular stress response triggered by endoplasmic reticulum (ER) stress, with the primary goal of restoring protein homeostasis in the ER lumen. This pathway is mediated by three sensor receptors that become activated under stress conditions: ATF6 (Activating Transcription Factor 6), IRE1α (Inositol-Requiring Enzyme 1α), and PERK (Protein kinase R-like ER Kinase) [8]. Active IRE1α processes the unspliced X-box binding protein 1 (*uXBP1*) mRNA, producing the active transcription factor spliced XBP1 (sXBP1), which regulates genes involved in protein folding and ER-associated degradation [9]. Concurrently, ATF6 translocates to the Golgi apparatus, where it undergoes proteolytic cleavage to generate cytoplasmic fragment ATF6f, an active transcription factor that modulates the expression of UPR target genes, including XBP1 [10]. Phosphorylated eIF2α (eukaryotic translation initiation factor 2α) is a key regulator of the third branch of the UPR and a central player in the integrated stress response (ISR). Its phosphorylation is governed by four serine/threonine kinases of the ISR: PERK, PKR (double-stranded RNA-dependent protein kinase), HRI (Heme-Regulated Inhibitor), and GCN2 (General Control Nonderepressible 2; *EIF2AK4*, Eukaryotic translation initiation factor 2-alpha kinase 4) [11]. Phosphorylated eIF2α causes a selective increase in the translation of specific mRNA, such as *ATF4* (Activating Transcription Factor 4) and a decrease in overall protein synthesis [12]. ATF4 induces the transcription of key stress response genes, notably *DDIT3* (DNA Damage-Inducible Transcript 3), which encodes CHOP (C/EBP homologous protein), a pro-apoptotic protein [5].

In cancer cells, ISR and UPR help manage stress, allowing cancer cells to survive and proliferate despite unfavorable conditions [13–16], which can also contribute to therapeutic resistance [17, 18]. However, when tumor cells are unable to restore homeostasis under chronic stress, sustained and prolonged activation of mechanisms such as the UPR and ISR leads to apoptosis, a process that could potentially be exploited to inhibit tumor growth [19–21].

Therefore, modulating ISR and UPR may offer novel strategies for sensitizing tumors to treatment and overcoming resistance, making it a significant focus in cancer biology and therapeutic development. Understanding the precise mechanisms by which NXP800 activates the UPR could lead to more effective and better-tailored therapeutic approaches. Here, we show that the small molecule NXP800 delays OS growth by activating UPR and ISR through the GCN2 kinase and subsequently the eIF2α/ATF4/CHOP pathway. This study strongly shows the therapeutic interest to activate chronic ER stress and UPR in OS.

## Results

### NXP800 decreases viability of OS cell lines by blocking proliferation and inducing apoptosis

**Fig. 1:**
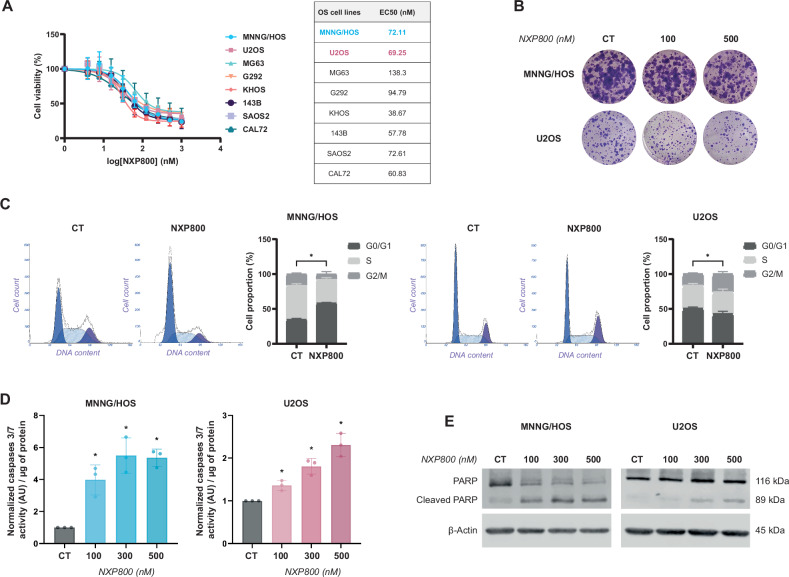
NXP800 decreases viability of OS cell lines by blocking proliferation and inducing apoptosis. **A** OS cell lines were treated with increasing concentrations of NXP800 (from 3.9 nM to 1 µM) for 72 h and cell viability was assessed by crystal violet assay. EC50s (nM) corresponding to each cell line were calculated with Graphpad prism (*N* = 3). **B** MNNG/HOS and U2OS cells were treated with NXP800 for 48 h and then reseeded without treatment for ten days to assess their ability to form colonies (*N* = 3). **C** MNNG/HOS and U2OS cells were treated with 300 nM NXP800 for 24 h and cell distributions in the different phases of the cell cycle were determined after PI staining and normalized to DMSO control condition (CT) (*N* = 4). **D** MNNG/HOS and U2OS cells were treated with NXP800 at indicated concentrations for 24 h. Caspase 3/7 activity (AU per µg protein) was evaluated on cell lysates and normalized to DMSO control condition (CT; *N* = 3). **E** MNNG/HOS and U2OS cells were treated with NXP800 at indicated concentrations for 24 h, and PARP cleavage was evaluated by western blotting (representative image of three independent biological replicates). In (**C**, **D**) bars indicate means ± SD of the different values, *: *p* < 0.05 (Mann-Whitney test).

First, the effect of NXP800 was investigated on the viability of eight OS cell lines representing genetic heterogeneity. NXP800 reduces the viability of OS cell lines in a dose-dependent manner after 72 h of treatment (Fig. 1A), while healthy human Mesenchymal Stem Cells (hMSCs) viability is slightly reduced (Fig. S1A). The EC50 values range between 40 and 140 nM, indicating a high sensitivity to NXP800 for all OS cell lines (Fig. 1A). Additionally, NXP800 reduces the clonogenic growth capacity of MNNG/HOS and U2OS cells after 48 h treatment with 100 and 500 nM (Fig. 1B). To explain this decrease in cell viability, cell cycle and apoptosis analyses were performed. NXP800 delays cell cycle in both cell lines. Indeed, NXP800 induces an accumulation of MNNG/HOS cells in the G0/G1 phase of the cell cycle (Fig. 1C, left panel), supporting by the increase of p21 and p27 expression and decrease of cyclin B1, cyclin D1, and CDC2 expression (Fig. S1B). A slight accumulation of U2OS cells in the G2/M phase is observed after 24 h of treatment (Fig. 1C, right panel), suggesting a slowdown in the cell cycle rather than a blockage. In addition, NXP800 induces a 4- to 5-fold increase in caspase-3/-7 activity compared to the control condition in MNNG/HOS cells, and a 1.2- to 2-fold increase in U2OS cells after 24 hours of treatment (Fig. 1D). These results were confirmed by an increase of PARP cleavage in a dose-dependent manner in both cell lines (Figs. 1E and S1C).

### NXP800 induces UPR through eIF2α phosphorylation

**Fig. 2:**
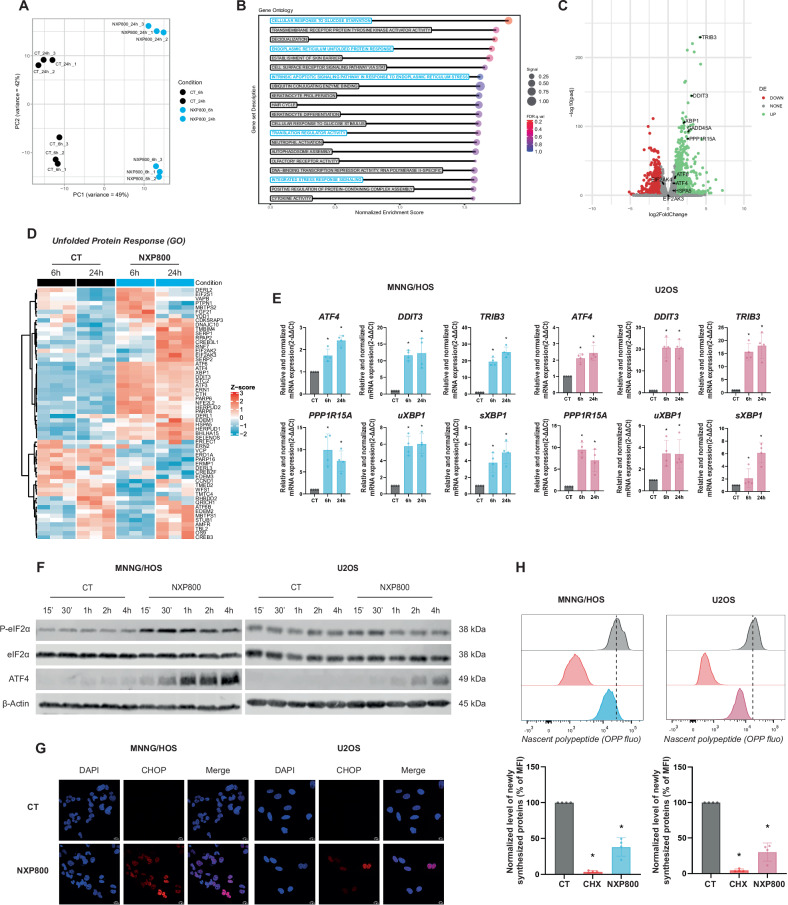
NXP800 induces UPR through eIF2A phosphorylation. **A** RNA-seq was performed on MNNG/HOS cells treated with DMSO (CT) or 300 nM NXP800 for 6 h and 24 h (*N* = 3). The Principal Component Analysis (PCA) plot illustrates the variance among different samples. Each point represents an individual sample, with clustering indicating similarities in global gene expression profiles across conditions. **B** GSEA was performed on Gene Ontologies (GO) biological processes with the genes ordered by their expression on MNNG/HOS cells between the treatment with 300 nM NXP800 for 6 h and the corresponding control. **C** The volcano plot illustrates the distribution of differentially expressed genes on RNA-seq data between the NXP800 treatment for 6 h and the corresponding control. **D** The heatmap of differentially expressed genes associated with the UPR pathway (GO) was generated from RNA-seq data. **E** MNNG/HOS and U2OS cells were treated with 300 nM NXP800 for 6 and 24 h. Relative mRNA expression of UPR genes was evaluated by RT-qPCR and normalized to DMSO control condition (CT; *N* = 4). **F** MNNG/HOS and U2OS cells were treated with 300 nM NXP800 for the indicated durations. Proteins expression the UPR pathway were evaluated by western blot (representative images of three independent biological replicates). **G** MNNG/HOS and U2OS cells were treated with 300 nM NXP800 for 4 h treatment and immunofluorescent labeling of CHOP and nucleus (DAPI) was performed (representative images of three independent biological replicates). **H** MNNG/HOS and U2OS cells were treated with 10 µM CHX used as a reference translation inhibitor or 300 nM NXP800. Translation rate was determined by OPP assay and normalized to DMSO (CT). In (**E**, **H**), bars indicate means ± SD of the different values, *: *p* < 0.05 (Mann-Whitney test).

To identify the molecular mechanisms underlying the effects of NXP800 on OS cell viability, RNA sequencing on MNNG/HOS cells was performed after 6 h and 24 h of treatment with 300 nM NXP800 compared to control conditions. First, the PCA plot shows high variance between the different tested conditions, particularly between the 6 and 24 h of NXP800 treatments, reflecting significant transcriptional differences among these conditions (Fig. 2A). Since NXP800 was initially designed for its ability to inhibit the HSF1 pathway, its effects on key components of the HSR were investigated. While the heatmap of transcript profiles associated with HSR reveals a downregulation of approximately two-thirds of these transcripts (Fig. S2A), surprisingly, NXP800 does not affect HSF1 or HSP70/72 expression at protein level after 24 h of treatment (Fig. S2B). However, gene set enrichment analysis (GSEA) based on Gene Ontology (GO) biological processes, after 6 h of treatment with NXP800 compared to control, indicates that the UPR and ISR pathway, the regulation of intrinsic apoptosis in response to ER stress and the translation regulator activity are strongly differentially upregulated (Fig. 2B). Additionally, NXP800 significantly induces multiple differentially expressed genes, including key targets of UPR activation such as *DDIT3* (encoding CHOP), *PPP1R15A* (encoding GADD34), *TRIB3*, and *XBP1*, after 6 h of treatment compared with control DMSO (Fig. 2C). The heatmap of expression profiles for transcripts associated with the UPR, as defined by GO, shows that approximately one-third of these transcripts exhibit increased expression after 6 h of treatment, while two-thirds show increased expression after 24 h, relative to the control in MNNG/HOS cells (Fig. 2D). The increased expression of UPR target genes, including *ATF4*, *DDIT3*, *TRIB3*, *GADD34*, *uXBP1*, and *sXBP1*, identified by RNA-seq, was validated in MNNG/HOS and U2OS cell lines using RT-qPCR (Fig. 2E). Moreover, NXP800 induces eIF2α phosphorylation after 15 min of treatment, while total eIF2α expression remains unchanged in MNNG/HOS and U2OS cells, as shown by western blotting analyses (Figs. 2F and S2C). Added to this, NXP800 increases ATF4 protein expression in a time-dependent manner in both cell lines (Figs. 2F and S2C). Consequently, CHOP is expressed in the nucleus of MNNG/HOS and U2OS cells treated with 300 nM NXP800 for 4 h, but is absent under the control condition (Fig. 2G). P-eIF2α is also known to reduce global protein synthesis, therefore, the capacity of cells to synthesize nascent proteins was assessed using the incorporation OPP assay. Cycloheximide (CHX), a specific inhibitor of protein translation, suppresses protein synthesis by 97%, confirming the efficiency of the assay (Fig. 2H). NXP800 significantly reduces protein synthesis by approximately 62% in MNNG/HOS cells and by 70% in U2OS cells, relative to the control (Fig. 2H).

### NXP800 engages UPR and ISR via GCN2 kinase activation

**Fig. 3:**
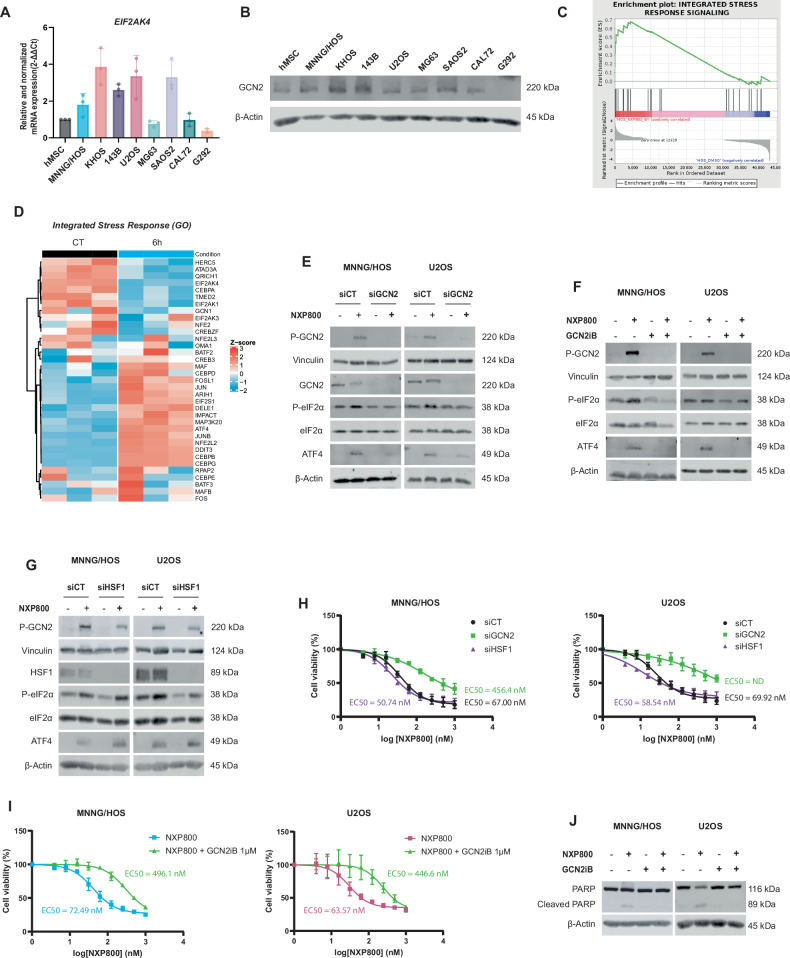
NXP800 engages UPR and ISR via GCN2 kinase. **A** The mRNA expression of *EIF2AK4* in OS cell lines compared to hMSCs was evaluated by RT-qPCR (*N* = 3) and **B** the protein expression of GCN2 was evaluated by western blot (representative images of two independent biological replicates). **C** The enrichment plot of ISR signaling was generated from RNA-seq data of MNNG/HOS treated with 300 nM NXP800 for 6 h, and **D** the corresponding heatmap illustrates differentially expressed genes, based on GO ontology. **E** MNNG/HOS and U2OS cells were transfected with siCT or siGCN2 for 40 h and then treated with DMSO (CT) or 300 nM NXP800 for 4 h. Expression of key ISR proteins was evaluated by western blot (representative images of three independent biological replicates). **F** MNNG/HOS and U2OS cells were treated with or without 1 µM GCN2iB for 24 h, and then with DMSO or 300 nM NXP800 for 4 h. Expression of key ISR proteins was evaluated by western blot (representative images of three independent biological replicates). **G** MNNG/HOS and U2OS cells were transfected with siCT or siHSF1 for 48 h and then treated with DMSO or 300 nM NXP800 for 4 h. Expression of key ISR proteins was evaluated by western blot (representative images of three independent biological replicates). **H** MNNG/HOS and U2OS cells were transfected with siCT, siHSF1 or siGCN2 for 24 h and then reseeded and treated with increasing concentrations of NXP800 (from 3.9 nM to 1 µM) for 72 h. Cell viability was assessed by crystal violet assay. EC50s (nM) corresponding to each cell line were calculated with Graphpad prism (*N* = 3). **I** MNNG/HOS and U2OS cells were treated with increasing concentrations of NXP800 (from 3.9 nM to 1 µM) in combination or not with 1 µM GCN2iB for 72 h, the cell viability was assessed by crystal violet assay. EC50s (nM) corresponding to each cell line were calculated with Graphpad prism (*N* = 4). **J** MNNG/HOS and U2OS cells were treated with NXP800 in combination or not with 1 µM GCN2iB for 24 h. Expression of PARP and cleaved PARP was evaluated by Western blot (representative images of three independent biological replicates). In (**A**, **H**, **I**), bars indicate means ± SD of the different values.

As NXP800 induces eIF2α phosphorylation, the kinase responsible for this effect among the four known eIF2α kinases was investigated. GSEA reveals enrichment of the cellular response to glucose starvation (Fig. 2B), pointing to a potential involvement of the ISR kinase GCN2 in the response to NXP800. Initially, the expression level of GCN2, encoded by *EIF2ΑK4*, was evaluated in various OS cell lines compared to healthy hMSCs. MNNG/HOS, KHOS, 143B, and SAOS2 cell lines exhibit higher *EIF2ΑK4*/GCN2 expression compared to U2OS, MG63, CAL72, G292, and hMSC cells (Fig. 3A, B). Moreover, *EIF2AK4* appears to be highly expressed in various cancers, including OS (Fig. S3A) and exhibits elevated expression in OS compared to healthy tissues, except in fibroblasts (Fig. S3B). Interestingly, MNNG/HOS cells treated with 300 nM of NXP800 exhibit enrichment of the ISR ontology compared to the control condition (Figs. 2B and 3C). Specifically, the expression of genes involved in the ISR, as defined by GO, is differentially regulated in 6 h NXP800-treated condition compared to control condition (Fig. 3D). We first confirmed that NXP800 activates GCN2, as indicated by increased GCN2 phosphorylation (Figs. 3E, F, and S4A, B). GCN2 phosphorylation is not induced by NXP800 when GCN2 is inhibited using siRNA or pharmacological approach using an ATP-competitive inhibitor of GCN2, GCN2iB (Fig. 3E, F). In addition, silencing GCN2 using siRNA prevents NXP800 from inducing eiF2α phosphorylation and ATF4 expression (Figs. 3E and S4A), which is confirmed by pharmacological inhibition of GCN2 (Figs. 3F and S4B). Furthermore, NXP800 continues to induce phosphorylation of both GCN2 and eiF2α, and consequently ATF4 expression when HSF1 is downregulated (Figs. 3G and S4C), showing that this pathway is independent of HSF1. Furthermore, GCN2 knockdown or inhibition markedly increases the EC50 values by 7-fold compared to respective control in MNNG/HOS and U2OS cells, while HSF1 knockdown has no impact (Fig. 3H, I), suggesting a critical role of GCN2, independent of HSF1, in the cytotoxic activity of NXP800. Notably, after several days of siHSF1 transfection, 70–90% of cells remained viable, compared to only 30–40% with siGCN2 (Fig. S4D). Consistently, NXP800 induces PARP cleavage, which is abrogated by GCN2 inhibition with GCN2iB (Figs. 3J and S4E), confirming that GCN2 activation is essential for inhibitory effects of NXP800. Moreover, using SPR experiments, dose-dependent binding responses are observed, indicating an interaction between NXP800 and GCN2-GST recombinant protein. The calculated KD value for the interaction is 4.8 × 10^−5^ M, indicating a low-affinity binding (Fig. S4F).

### NXP800 induces IREα/JNK/c-Jun pathway

**Fig. 4:**
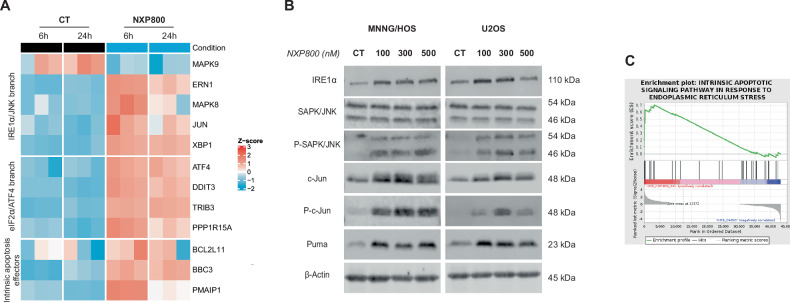
NXP800 induces IREα/JNK/c-Jun pathway. **A** The heatmap of selected differentially expressed genes was produced from RNA-seq data of MNNG/HOS treated with 300 nM NXP800 or DMSO (CT) for 6 and 24 h. **B** MNNG/HOS and U2OS cells were treated with indicated concentrations of NXP800 or DMSO (CT) for 24 h. The expression of proteins involved in IRE1a/JNK signaling and apoptosis was assessed by western blot (representative images of three independent biological replicates). **C** The enrichment plot of “Intrinsic apoptotic signaling pathway in response to ER stress” based on GO was generated from RNA-seq data.

It is known that JNK signaling cascade is directly linked to UPR activation via IRE1α [22]. RNA-seq analysis reveals the overexpression of key genes involved in the IRE1α/JNK/c-Jun signaling pathway and apoptosis including *ERN1* (encoding IRE1α), *JUN* (encoding c-Jun), and *MAPK8* (encoding JNK) (Fig. 4A). Interestingly, after 24 h of treatment with increasing doses of NXP800, the total protein levels of SAPK/JNK remain unchanged, while its phosphorylated form is increased in both MNNG/HOS and U2OS cells (Figs. 4B and S5A). Similarly, both phosphorylated and total c-Jun levels are increased after NXP800 treatment compared to control DMSO (Figs. 4B and S5A). Additionally, RNA-seq analysis revealed an enrichment of the intrinsic apoptosis signaling in response to ER stress (Fig. 4C), and *BBC3*, encoding the pro-apoptotic protein Puma, is upregulated at both the transcriptional and protein levels after NXP800 treatment in both cell lines compared to control condition (Figs. 4A, B and S5A).

### NXP800 delays tumor growth in preclinical OS xenograft model

**Fig. 5:**
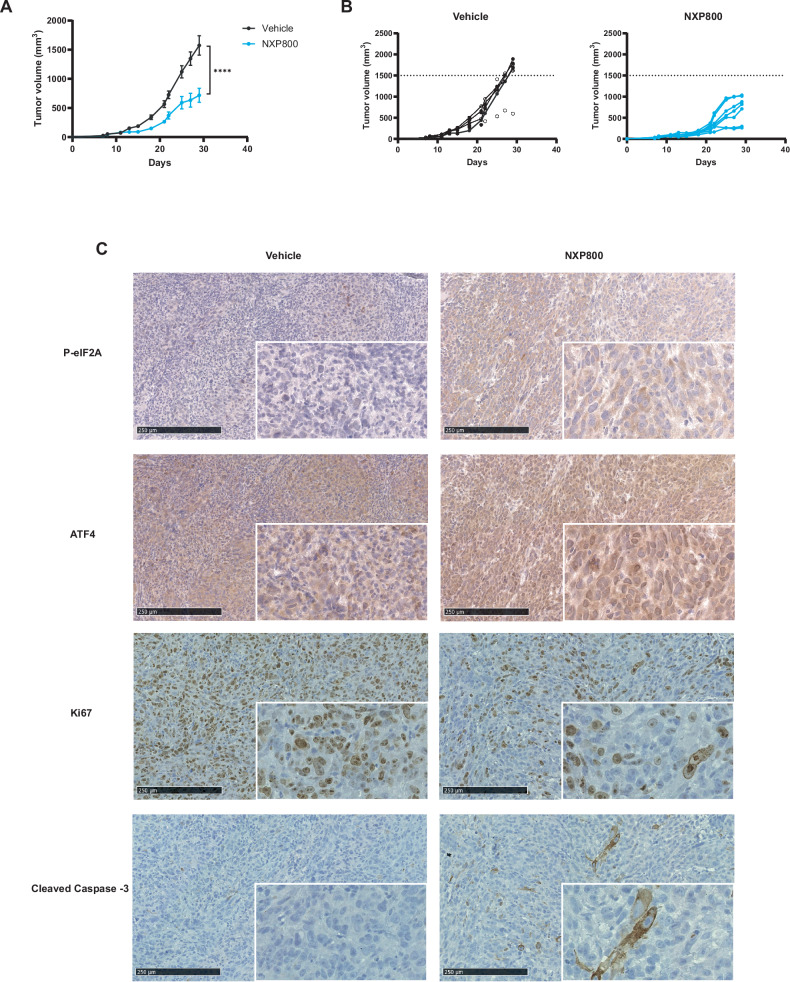
NXP800 delays tumor growth in preclinical OS xenograft model. **A, B** One million MNNG/HOS cells were injected into the para-tibial region of NMRI nude mice. Once tumors were palpable, mice were randomized and orally treated with vehicle or 35 mg/kg NXP800 5 days per week. Tumor volumes were measured three times a week until sacrifice (vehicle group, *N* = 7; NXP800-treated group, *N* = 7) and represented as (**A**) mean tumor volume or (**B**) individual tumor volume. **C** Immunohistochemistry staining of P-eiF2α, ATF4, Ki-67, and cleaved caspase-3 was performed on tumor sections from mice grafted with 2.10⁶ MNNG/HOS cells and treated daily for 5 days only (Magnification x10 and zoom x40). In (**A**, **B**), bars indicate means ± SEM of the different values, ****: *p* < 0.001 (Anova test).

To assess the in vivo efficacy of NXP800, NMRI nude mice were xenografted with 1.10⁶ MNNG/HOS cells in the para-tibial region and randomized into two groups (*N* = 7 per group). The mice were orally treated with 35 mg/kg NXP800 or vehicle, with the regimen adjusted as needed to allow recovery from weight loss observed in NXP800-treated mice (Fig. S6A, B). NXP800 significantly delays tumor growth (*p* < 0.001), with treated mice reaching an average tumor volume of 700 mm³ at the endpoint, compared to approximatively 1500 mm³ in the vehicle-treated group (Fig. 5A, B). Consistent with in vitro data, tumors of NXP800-treated mice exhibit higher eIF2α and ATF4 expression compared with the control group, as shown by immunohistochemical analysis in tumors from mice treated daily for 5 days (*N* = 4 per group; Fig. 5C). In addition, NXP800 decreases Ki-67 expression, a marker of proliferating cells, with an average of 61.12% positive nuclei in the control group compared to 42.26% in the treated group, (*N* = 4 per group; Figs. 5C and S6C). NXP800 induces apoptosis, as shown by an almost three-fold increase in cleaved caspase-3 expression in the treated group compared to the vehicle group (Figs. 5C and S6C). Notably, one treated mouse responded less well to treatment, showing higher Ki-67 positivity (61.9%) and lower cleaved caspase-3 positivity (1.43%; Fig. S6C).

To assess systemic toxicity, tumor-free NMRI mice were treated orally with NXP800 (35 mg/kg, 5 days/week for 2 weeks; *N* = 5 per group). Serum ALT and AST levels is significantly decreased in the treated group (Fig. S6D) and lung and liver weights were lower in the treated group, while the other organs appeared unaffected (Fig. S6E). Histopathological analysis revealed no significant lesions in any organ of the treated group compared to the control group.

## Discussion

OS is the most common primary malignant bone tumor in children and adolescents. Despite actual treatment strategies combining surgery and chemotherapy, therapeutic outcomes have remained unchanged for decades, highlighting the need for novel targeted therapies. Among them, NXP800, a novel orally available compound, was developed as an inhibitor of the HSF1 pathway based on its ability to reduce HSP72 expression in ovarian cancer [6], and is currently undergoing a Phase 1 clinical trial for advanced ovarian cancer (NCT05226507) and cholangiocarcinoma (NCT06420349). Indeed, HSF1 a key player in the HSR, plays a multifaceted role in key cancer processes, including survival, proliferation, metabolism, protein synthesis, migration, and invasion [4, 23, 24]. In OS, HSF1 is a target of interest since it supports malignancy through its involvement in proliferation, survival, and migration [25]. In this context, we investigated the therapeutic potential of NXP800 in OS.

Our study shows that NXP800 decreases the viability of several OS cell lines as well as their clonogenic capacity following treatment, demonstrating a strong anti-tumor effect in vitro. These effects are explained by both a proliferation blockade and an induction of apoptosis. To better understand NXP800’s mechanisms of action, RNA-seq analysis allowed to identify direct targets of NXP800 and also to explore signaling pathways modulated over the long term. As expected, RNA-seq analysis revealed a slight modulation of the HSR, but we failed to confirm this at the protein level. Nevertheless, the decrease in HSP72 expression observed with NXP800 in ovarian cancer or in prostate cancer occurred following stimulation of the HSF1 pathway with an HSP90 inhibitor [6, 7], which was not performed in our study. However, RNA-seq data revealed a strong increase in the UPR signature in cells treated with NXP800. Indeed, NXP800 induces the transcription of eIF2α/ATF4 target genes such as *DDIT3, PPP1R15A*, and *TRIB3*. This suggests that NXP800 triggers the UPR, as evidenced by the phosphorylation of eIF2α, which leads to both a global reduction in protein synthesis and an increased expression of the transcription factor ATF4 along with its downstream target CHOP (encoded by *DDIT3*). Furthermore, NXP800 also induces the expression of *uXBP1* and *sXBP1* mRNAs, suggesting the activation of both the ATF6 and IRE1α axis, as ATF6 promotes *uXBP1* transcription, which is subsequently spliced by IRE1α into *sXBP1* [8]. In advanced treatment-resistant prostate cancer, similar results were observed, as NXP800 induces all three axis of the UPR (PERK/eIF2α, ATF6, and IRE1α), ultimately inhibiting tumor growth [7].

To investigate the mechanisms driving eIF2α phosphorylation, we focused on GCN2, one of four kinases with PERK, HRI, and PKR regulating eIF2α axis in the ISR [26]. By detecting uncharged tRNA levels, GCN2 plays a crucial role in the cellular stress response, particularly under conditions of amino acid deprivation, influencing cell growth, survival, and metabolism through the activation of the ISR [21]. First of all, RNA-seq analysis confirmed that NXP800 induces the ISR in tumor cells. We validated that NXP800 activates the GCN2 kinase as a novel molecular target by inducing its phosphorylation independently of the HSF1 pathway. By inhibiting GCN2 using molecular and chemical approaches, we confirmed that NXP800 no longer induces eIF2α phosphorylation or increases ATF4 expression. Additionally, inhibition of GCN2 reverses the effect of NXP800 on the inhibition of tumor cell viability, showing that NXP800 induces ISR and subsequently UPR via GCN2 kinase. Moreover, we observed an interaction between NXP800 and the recombinant GCN2-GST protein with low binding affinity, indicating that further experiments are needed to determine the binding site of NXP800 on GCN2 and rule out any interaction with the GST tag. Recent studies highlight the complex and dual role of GCN2 in cancer, given its involvement in ISR and UPR [16, 27, 28]. GCN2 plays a protective role in tumor cells, allowing them to survive in a hostile and stressful environment, but can also induce cell death when its activation is sustained and prolonged [21]. This dual role also applies to UPR, which, when prolonged and cells fail to restore homeostasis, switches from a pro-survival effect to a program that promotes apoptosis [19]. Furthermore, GCN2-mediated apoptosis occurs through the eIF2α/ATF4/CHOP pathway [29], which we confirmed to be strongly induced with NXP800. However, to date, no study has reported the involvement of GCN2 in OS, making our findings particularly novel and interesting in this context.

To better understand the signaling pathways by which NXP800 reduces OS proliferation and induces apoptosis, we demonstrated that NXP800 induces the IRE1α/JNK/c-Jun pathway, a key signaling cascade in cancer and OS [30, 31]. Concurrently, IRE1α expression increases, and both JNK and c-Jun are phosphorylated following NXP800 treatment. The activation of the JNK pathway, known to play a role in apoptosis [32], is supported by increased expression of Puma, a pro-apoptotic protein in the intrinsic pathway. Our data suggest that NXP800 activates IRE1α/JNK/c-Jun axis of UPR leading to the induction of Puma expression.

In line with our in vitro data, we demonstrated for the first time that NXP800 delays tumor growth in a preclinical mouse OS xenograft model by activating UPR, decreasing cell proliferation and inducing caspase-3-mediated apoptosis. Consistent with our results, the GCN2 activator molecule HC-7366 was described to inhibit tumor growth in murine xenograft models of fibrosarcoma and acute myeloid leukemia [33]. In vitro, HC-7366 appears to enhance the transcriptional activity of ATF4 and c-Jun while inducing the pro-apoptotic protein Puma, confirming our results [34].

Taken together, our data demonstrate for the first time the therapeutic potential of NXP800 to inhibit OS tumor growth by activating ISR and UPR, in accordance with recent studies in other cancers [35, 36]. Indeed, in a first phase I clinical trial, NXP800 showed promising pharmacokinetics and tolerable dosing schedule with biomarker modulation including transcriptional targets of eIF2α/ATF4 axis such as *NUPR1*, *ULBP1*, and *TRIB3* (NCT05226507), further confirming its mechanism of action. Preclinical toxicity results are consistent with the safety profile reported in the ongoing phase I clinical trial of NXP800 in patients with advanced ovarian cancer (NCT05226507), where treatment-related adverse events were mostly grade 1–2 gastrointestinal and metabolic (mainly nausea and vomiting), while increases in AST and ALT were rare and low-grade [37]. In clinical perspective, it would be of interest to investigate the synergistic effect of NXP800 in combination with conventional chemotherapies, which have been reported to induce eIF2α phosphorylation via GCN2 and PKR in melanoma cells [38]. Thus, GCN2 emerges as a promising therapeutic target in OS and potentially in other tumors as well [39].

In conclusion, this study provides the first preclinical evidence that NXP800 delays OS tumor growth by activating the ISR and UPR through GCN2 kinase. We demonstrate that NXP800 induces eIF2α phosphorylation, ATF4 upregulation, and pro-apoptotic signaling, leading to reduced OS cell viability in vitro and significant tumor growth inhibition in a preclinical xenograft model. Our findings establish for the first time a novel therapeutic mechanism for NXP800 through the GCN2 activation, independent of its previously known effects on HSF1. These findings suggest that targeting stress response pathways, particularly GCN2-mediated ISR and UPR activation could represent a new therapeutic strategy for OS. Given that NXP800 is currently in clinical trials for other cancers, these results support its potential as therapeutic option in OS treatment, either as a monotherapy or in combination with existing chemotherapies.

## Materials and methods

### Cell culture

Human OS cell lines used (MNNG/HOS, U2OS, MG63, G292, KHOS, 143B, SaOS2, CAL72) were purchased from the American Type Culture Collection (ATCC) and authenticated by STR profiling. They were cultured in DMEM High Glucose (Biosera) supplemented with 10% fetal bovine serum (FBS; Eurobio) at 37 °C and 5% CO_2_. Cells were tested negative for mycoplasma using PCR. Human mesenchymal stem cells (hMSCs) from healthy donors (IKT Ulm) were cultured in α-MEM (Thermo Fisher Scientific) supplemented with 5% platelet lysate and plasma (PLP; IKT Ulm), heparin (1 UI/mL; Panpharma), and 1% penicillin/streptomycin (Biosera).

### Therapeutic agent

The clinical candidate CCT36184 has been licensed by Nuvectis Pharma, Inc. as NXP800 and was kindly provided by Nuvectis Pharma, Inc. NXP800 was dissolved in DMSO at 10 mM. GCN2 inhibitor, GCN2iB (HY-112654), is purchased from MedChemExpress. GCN2iB was dissolved in DMSO at 20 mM and was used at a concentration of 1 µM alone or in combination with NXP800.

### Cell viability

OS cell lines were seeded in 96-well plates and then treated for 72 hours with a range of increasing concentrations of NXP800 (from 3.9 nM to 1 µM, ½ fold cascade dilution). Cells were fixed with 1% glutaraldehyde (Sigma, MA, USA) and then stained with 0.01% crystal violet (Sigma), which was then dissolved with Sorensen’s solution (trisodium citrate, 0.1 N HCl, and ethanol). Absorbance was read with a spectrophotometer (Perkin Elmer Wallac 1420 Victor2). Each range point was normalized to DMSO control condition.

### Clonogenic capacity assay

Cells were treated for 48 h with DMSO or NXP800 (100 and 500 nM) and then reseeded at 1000 cells per well in 6-well plates in untreated medium for 10 days. Cells were fixed with 1% glutaraldehyde (Sigma) and then stained with 0.01% crystal violet (Sigma).

### Cell cycle analysis

Cells were treated with DMSO or NXP800, rinsed with PBS, fixed with 70° ethanol, buffered with phospho-citrate buffer and incubated with 1 µg/mL RNAse A (QIAGEN). Cells were then labeled with 50 µg propidium iodide (PI) and fluorescence intensity was read by FACSymphony A5 (BD Biosciences). The cell cycle was determined using Multicycle AV Cell Cycle software.

### Caspases -3/-7 activity assay

Cells treated with DMSO or NXP800 were lysed using RIPA (Tris-HCl 50 mM, NaCl 150 mM, NP40 1%, SDC 0.25%, Sodium fluoride 1 mM) supplemented with protease inhibitor cocktail (Roche), phenylsulfonyl fluoride and Na_3_VO_4_ to preserve proteins. Caspase -3 and -7 activity was assessed on Tristar LB (Berthold Technologies) using the Apo-ONE Homogeneous Caspase -3/-7 Assay kit (Promega), following the manufacturer’s instructions. Results are presented in arbitrary units (AU) per µg of protein.

### Cell transfection

Transfection was performed on cells at 60% confluence. Lipofectamine RNAiMAX (Invitrogen) was used as a transfection agent according to the manufacturer’s recommendations. siGCN2 (sc-45644, Santa Cruz Biotechnology), siHSF1 (sc-35611, Santa Cruz Biotechnology) and control siRNA (4457287, Ambion) were transfected at a final concentration of 30 nM for 40 or 48 h depending of the siRNA.

### Western blot

Cells were treated at different concentrations and times with DMSO, NXP800 or GCN2iB, then lysed to extract proteins with 5% SDS followed by sonication. Proteins were then denatured for 5 min at 95 °C in Laemmli buffer (31 mM Tris pH 6.8; 0.5% SDS; 5% glycerol; 1.25% B-mercaptoethanol; 0.005% bromophenol blue). 30–60 µg of proteins were separated by electrophoresis in an acrylamide gel, then transferred to a PVDF membrane (Merck Millipore). The membrane was then saturated for 1 h with TBS Odyssey Blocking Buffer (LI-COR) and incubated overnight with primary antibodies (Table S1). Fluorescence revelation was performed after incubation with the following secondary antibodies: IRDye 680RD Donkey anti-Rabbit IgG (926-68073, LI-COR), IRDye 800CW Donkey anti-Mouse IgG 926-68072, LI-COR), using LI-COR Odyssey Fc (LI-COR). Protein expression was quantified by Western blot densitometry using Image Studio software. Signals were normalized to the respective loading control (β-Actin or Vinculin, depending on target protein molecular weight) and expressed relative to control.

### Immunofluorescence

Cells were seeded in eight well ibiTreat µ-slide (ibidi), then treated with DMSO or NXP800 for 4 h. Cells were fixed with paraformaldehyde 1%, permeabilized with 0.3% PBS-Triton (Sigma), saturated with normal goat serum 5% and incubated overnight with anti-CHOP primary antibody (Table S1). After washing, cells were incubated with the fluorescent anti-mouse antibody (A11032, Life Technologies), then with DAPI (D9542, Sigma) and mounted with mounting medium (ibidi). Images were acquired with Nikon Ti2 LFOV A1Rs microscope and processed with ImageJ software.

### RNA-sequencing

RNA sequencing was carried out on MNNG/HOS cell line, with either DMSO or NXP800 at 300 nM. RNA was extracted after 6 h and 24 h of treatment with 300 nM of NXP800. Each condition contains three replicates. Library was prepared by BGI Genomics with DNBSEQ Eukaryotic Strand-specific mRNA library kit, and was sequenced with DNBSEQ-G400 in a 2 × 100 bp (PE100) format. Reads were aligned on hg38 version “analysis set” with the RefSeq annotation “GCF_000001405.40”. For the Gene Set Enrichment Analysis (GSEA), the GSEA tool was used on human genes from Gene Ontology (GO) ontologies. The packages used for the analyses are detailed in Table S2.

### Other databases

Cancer Cell Line Encyclopedia (CCLE, https://sites.broadinstitute.org/ccle/) database was used to compare *EIF2ΑK4* expression between tumor cell lines (CCLE—DepMap 22Q4) and healthy tissues (GTEx version 8).

### RT-qPCR

Total RNA from cells treated with DMSO or NXP800 was extracted with the NucleoSpin RNA Plus kit (Macherey Nagel) following the manufacturer’s instructions. The mRNA was retrotranscribed to cDNA with the Maxima H Minus First Strand cDNA Synthesis kit (Thermo Scientific) at a concentration of 10 ng/µL. qPCR was performed using Master Mix Select SYBR (Applied Biosystems) and QuantStudio™ 7 Flex Real-Time PCR System (Applied Biosystems). The comparative cycle threshold method was used to calculate the relative expression of target mRNAs, and results were normalized to the expression of invariant reference genes Hypoxanthine Phosphoribosyltransferase (*HPRT*) and Beta-2-Microglobulin (*B2M*). The list of primers used is detailed in Table S3.

### OPP

Protein Synthesis Assay Kit (ab239725, Abcam) was used for the detection of global nascent proteins of OS cell lines following the instructions of the manufacturer. Briefly, cells were treated for 3 h with DMSO, 10 µM cycloheximide (CHX; Sigma) or 300 nM NXP800, including 2 h incubation with protein label O-propargyl-puromycin (OPP). After fixation and permeabilization, the reaction cocktail was added and incubated with the cells away from light for 30 min. After washing with PBS, the fluorescence was quantified by flow cytometry on a FACSymphony A5 (BD Biosciences) and Mean Fluorescence Intensity (MFI) was analyzed with FlowJo.

### Surface plasmon resonance (SPR) experiments

SPR measurements were performed by the Imp@ct core facility (SFR bonamy, Nantes Université, CNRS, Inserm, Nantes, France) to characterize the interaction between the chemical compound of interest NXP800 and recombinant human protein GCN2 fused to glutathione S-transferase (GCN2–GST; #14-934, Eurofins DiscoverX). Experiments were carried out on a Biacore T200 instrument using CM5 sensor chips (Cytiva). Prior to protein capture, the surface of the CM5 chip was functionalized with an anti-GST antibody according to the manufacturer’s guidelines, using standard amine-coupling chemistry. Following immobilization of the anti-GST antibody, GCN2–GST was injected at a concentration of 400 nM and captured onto the surface, yielding approximately 1500 response units (RU) of bound protein. A reference flow cell underwent the same coupling procedure without protein capture and was used for background subtraction. Binding analyses were performed using Single-Cycle Kinetics (SCK). The compound was injected at increasing concentrations of 0, 6.25, 12.5, 25, 50, and 100 µM, sequentially, over the immobilized GCN2–GST without regeneration between injections. Each cycle consisted of five injections with an association phase of 120 s, followed by a dissociation phase of 800 s. All experiments were conducted at 25 °C in running buffer (HBS-NP-1% DMSO).

### OS xenograft model

All experiments were carried out in an animal facility approved by the French Ministry of Agriculture for experimental use (Unité de Thérapeutique Expérimentale, Nantes; n°E44015). The protocol described in this project was approved by the local ethics committee CEEA-PdL06 of Nantes, project APAFIS #8405. This study was conducted in a xenograft model using 5-week-old female NMRI nude mice. MNNG/HOS cells (1.10^6^ cells/50 µl) were injected intramuscularly in contact with the tibia. Once tumor volume reached 100 mm^3^, animals were randomized into groups of seven mice and treated orally with vehicle (10% DMSO, 90% of 25% 2-hydroxypropyl-beta-cyclodextrin in 50 mM sodium citrate buffer pH 5) or 35 mg/kg NXP800, 5 times a week for 3–4 weeks (endpoint: tumor volume ≤2000 mm^3^). An additional experiment was conducted with a shorter treatment duration. Eight mice were grafted with 2.10⁶ MNNG/HOS cells and randomized into two groups of four mice when the tumor volume reached approximately 300 mm³. The mice were treated daily for five consecutive days, and tumors were collected for immunohistochemical analysis.

### In vivo toxicity evaluation

Tumor-free NMRI nude mice were randomized into two groups of five mice and orally treated with vehicle or NXP800 (35 mg/kg, 5 days/week for 2 weeks). After treatment, blood (intracardiac puncture) and organs (heart, lungs, liver, spleen, kidneys) were collected and examined in accordance with the revised guides for organ sampling and trimming in rats and mice [40–42]. Organs were subsequently fixed in formalin by immersion, embedded in paraffin, cut into 3 µm thick sections and stained with haemalun-eosin-saffron. The histopathological examination was performed by a veterinary pathologist certified by the European College of Veterinary Pathologists, Prof. Marie-Anne COLLE (Veterinary School of Nantes, France).

### ALT and AST ELISA

The serum from each mouse was diluted 1:400, and alanine aminotransferase (ALT) and aspartate aminotransferase (AST) levels were measured by ELISA according to the manufacturer’s instructions (kits ab282882 and ab263882, Abcam).

### Immunohistochemistry (IHC)

Collected tumors were fixed in 4% formaldehyde for 24 h. Serial 4 µm sections were cut at two levels. Slides were deparaffinized and antigen demasking were performed in 10 mM citrate buffer or Tris EDTA buffer (TP Module Leica). Slides were treated with H2O2 (Gifrer) to block endogenous peroxidase activity, then saturated with non-immune donkey serum 2%, BSA 1%, diluted in TBS tween 0.05% pH 7.4. Primary antibodies Ki-67, Cleaved Caspase-3, P-eIF2α and ATF4 (Table S1) were incubated for 2 h at RT. After rinsing, slides were incubated for 1 h at RT with biotinylated secondary antibody (E0432, Dako), and then incubated for 1 h at RT with streptavidin/peroxidase (P0397, Dako). Revelation was carried out with DAB (3, 3′-diaminobenzidine, F/ACK500, MM France). Slides were then counterstained with Gill-2 hematoxylin, dehydrated, cleared and mounted between slides and coverslips with Pertex. Staining and immunolabeling were acquired using a slide scanner (Nanozoomer, Hamamatsu). For each immunostain, images taken in a field representative of the tumor progression front were exported from the raw file at ×10 and ×40 magnification. Ki-67 and Cleaved Caspase-3 IHC were quantified using the QuPath software.

### Statistical tests

The results are presented as the mean of at least three independent experiments; the exact number of repetitions is indicated in the legend of each figure (N). Histogram and data are shown as mean ±SD or ±SEM. The western blot images shown are representative of experiments performed three times independently. Non-parametric (Mann-Whitney) and parametric (ANOVA) statistical tests were performed using Graphpad Prism software (GraphPad Software, Inc.). The risk α considered is 5% (*p < 0.05, ****p < 0.0001).

## Supplementary information

Supplemental materials

Supplemental figures

Uncropped WB

## Data Availability

The RNA-seq dataset supporting the conclusions of this article is available in the Gene Expression Omnibus repository (GEO) under accession number GSE283602. The computer code produced in this study is available on GitHub (https://github.com/EpistressLab/NXP800_osteosarcoma).
