# Supplementary material for: Activating GCN2 and subsequently the Unfolded Protein Response with the small oral molecule NXP800 delays tumor growth in osteosarcoma
Source: Cell Death Discov. 2026 Feb 5;12:94. doi: 10.1038/s41420-026-02941-2 (PMC12895007; doi:10.1038/s41420-026-02941-2)
Supplement: Supplementary file 1 — Supplemental materials [file 41420_2026_2941_MOESM1_ESM.docx]

**Table S1: List of antibodies used in western blot, immunofluorescence or immunohistochemistry.**

| **Target protein** | **Reference** | **Supplier** |
| --- | --- | --- |
| ATF4 (WB) | 11815 | Cell Signaling Technology |
| ATF4 (IHC) | 10835-A | Proteintech |
| B-Actin | 3700 | Cell Signaling Technology |
| c-Jun | 9165 | Cell Signaling Technology |
| Cyclin D1 | 2922 | Cell Signaling Technology |
| eIF2α | sc-133132 | Santa Cruz Biotechnology |
| Phospho-GCN2 Thr899 | ab75836 | Abcam |
| GCN2 | 3302 | Cell Signaling Technology |
| HSF1 | 4356 | Cell Signaling Technology |
| HSP70/72 (C92F3A-5) | ADI-SPA-810 | Enzo Life Sciences |
| IRE1a | 3294 | Cell Signaling Technology |
| SAPK/JNK | 9251 | Cell Signaling Technology |
| PARP | 9542 | Cell Signaling Technology |
| Puma | 98672 | Cell Signaling Technology |
| Phospho-c-Jun Ser63 | 9261 | Cell Signaling Technology |
| Phospho-eIF2α Ser51 (WB) | 3597 | Cell Signaling Technology |
| Phospho-eIF2α Ser51 (IHC) | ab32157 | Abcam |
| Phospho-SAPK/JNK Thr183/Tyr185 | 4668 | Cell Signaling Technology |
| CHOP | 2895 | Cell Signaling Technology |
| Ki-67 | M724001-2 | Agilent |
| Cleaved Caspase 3 | 9664 | Cell Signaling Technology |

**Table S2: Packages used for RNA-seq analysis.**

| **Package** | **Version** | **Citation** |
| --- | --- | --- |
| fastp | 0.20.1 | https://doi.org/10.1093/bioinformatics/bty560 |
| STAR | 2.7.5a | https://doi.org/10.1093/bioinformatics/bts635 |
| htseq_count | 0.12.4 | https://doi.org/10.1093/bioinformatics/btu638 |
| DESeq2 | 1.30.1 | https://doi.org/10.1186/s13059-014-0550-8 |
| ComplexHeatmap | 2.6.2 | https://doi.org/10.1002/imt2.43 |
| GSEA | 4.3.2 | https://doi.org//10.1038/ng1180 |

**Table S3: List of primers used for RT-qPCR.**

| **Gene target** | **Primers sequences** |
| --- | --- |
| ATF4_F | GTTCTCCAGCGACAAGGCTA |
| ATF4_R | ATCCTGCTTGCTGTTGTTGG |
| DDIT3_F | AGAACCAGGAAACGGAAACAGA |
| DDIT3_R | TCTCCTTCATGCGCTGCTTT |
| GADD34_F | TCCGACTGCAAAGGCGGCTCA |
| GADD34_R | CAGCCAGGAAATGGACAGTGAC |
| sXBP1_F | GCTGAGTCCGCAGCAGGT |
| sXBP1_R | CTGGGTCCAAGTTGTCCAGAAT |
| TRIB3_F | GCTTTGTCTTCGCTGACCGTGA |
| TRIB3_R | CTGAGTATCTCAGGTCCCACGT |
| uXBP1_F | CAGACTACGTGCACCTCTGC |
| uXBP1_R | CTGGGTCCAAGTTGTCCAGAAT |
| B2M_F | ttctggcctggaggctatc |
| B2M_R | tcaggaaatttgactttccattc |
| HPRT_F | tgaccttgatttattttgcatacc |
| HPRT_R | cgagcaagacgttcagtcct |
| GCN2_F | GTGAGCCATCAGTGACGACTGA |
| GCN2_R | AAAGCCTCCAGAGTCTGACGGT |
