# Supplementary material for: Activating GCN2 and subsequently the Unfolded Protein Response with the small oral molecule NXP800 delays tumor growth in osteosarcoma
Source: Cell Death Discov. 2026 Feb 5;12:94. doi: 10.1038/s41420-026-02941-2 (PMC12895007; doi:10.1038/s41420-026-02941-2)
Supplement: Supplementary file 2 — Supplemental figures [file 41420_2026_2941_MOESM2_ESM.pdf]

Figure S1

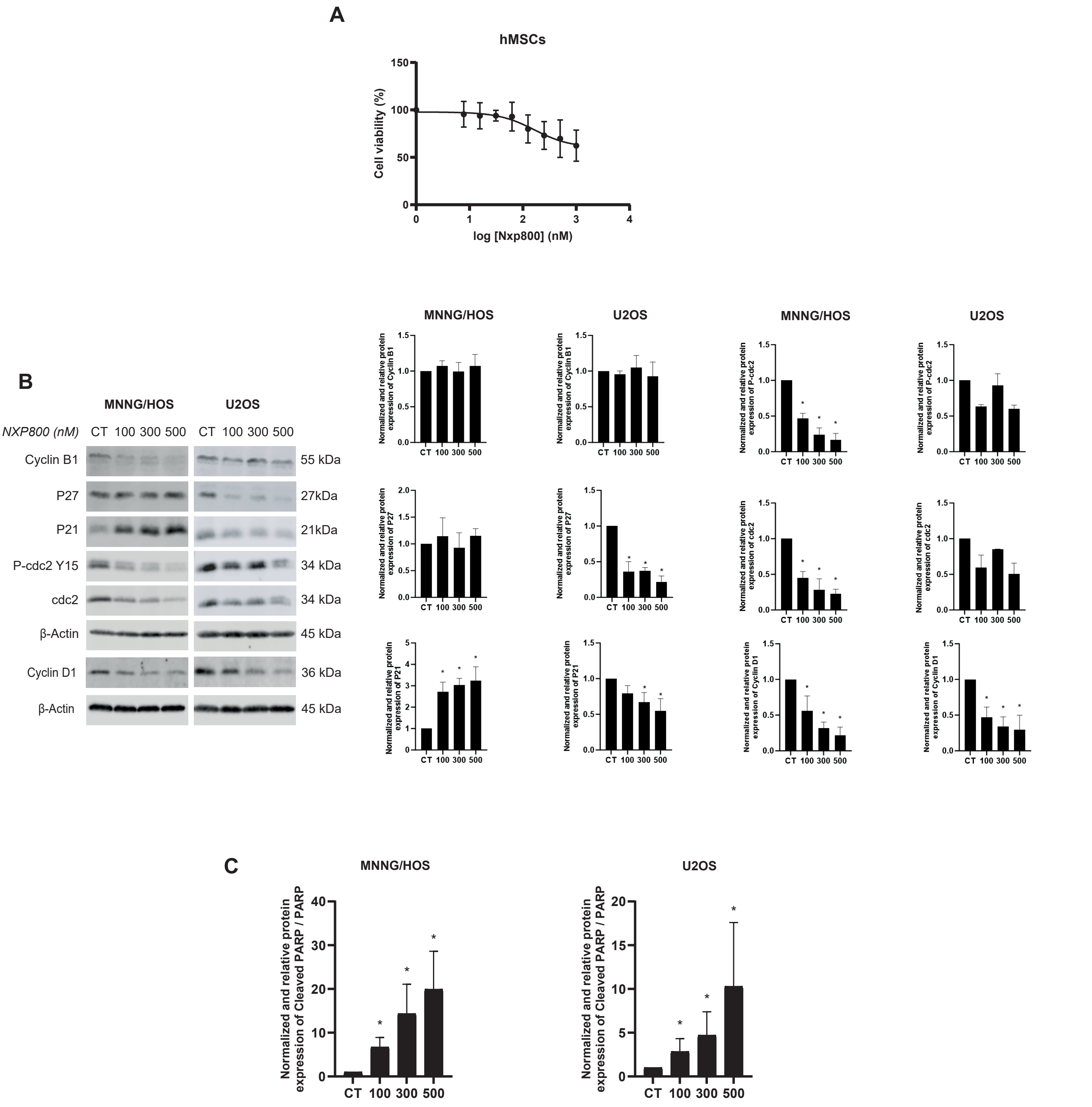

Figure S1: (A) Human mesenchymal stem cells (hMSCs) from healthy donors were treated with increasing concentrations of NXP800 (from 3.9nM to 1µM) for 72 hours and cell viability was assessed by crystal violet assay (N=3). (B) MNNG/HOS and U2OS cells were treated with indicated concentrations of NXP800 or DMSO (CT) for 24 hours. The expression of proteins involved in the cell cycle was assessed by Western blot (representative images of three independent experiments). Quantification of Western blot (N=3) was performed by densitometric analysis, normalized to β-Actin and expressed relative to CT. (C) Quantification of Western blot (N=3, from figure 1E) was performed by densitometric analysis, normalized to β-Actin and expressed relative to CT. In A, B and C, bars indicate means ± SD of the different values, *: $p < 0.05$ (Mann-Whitney test).

Figure S2

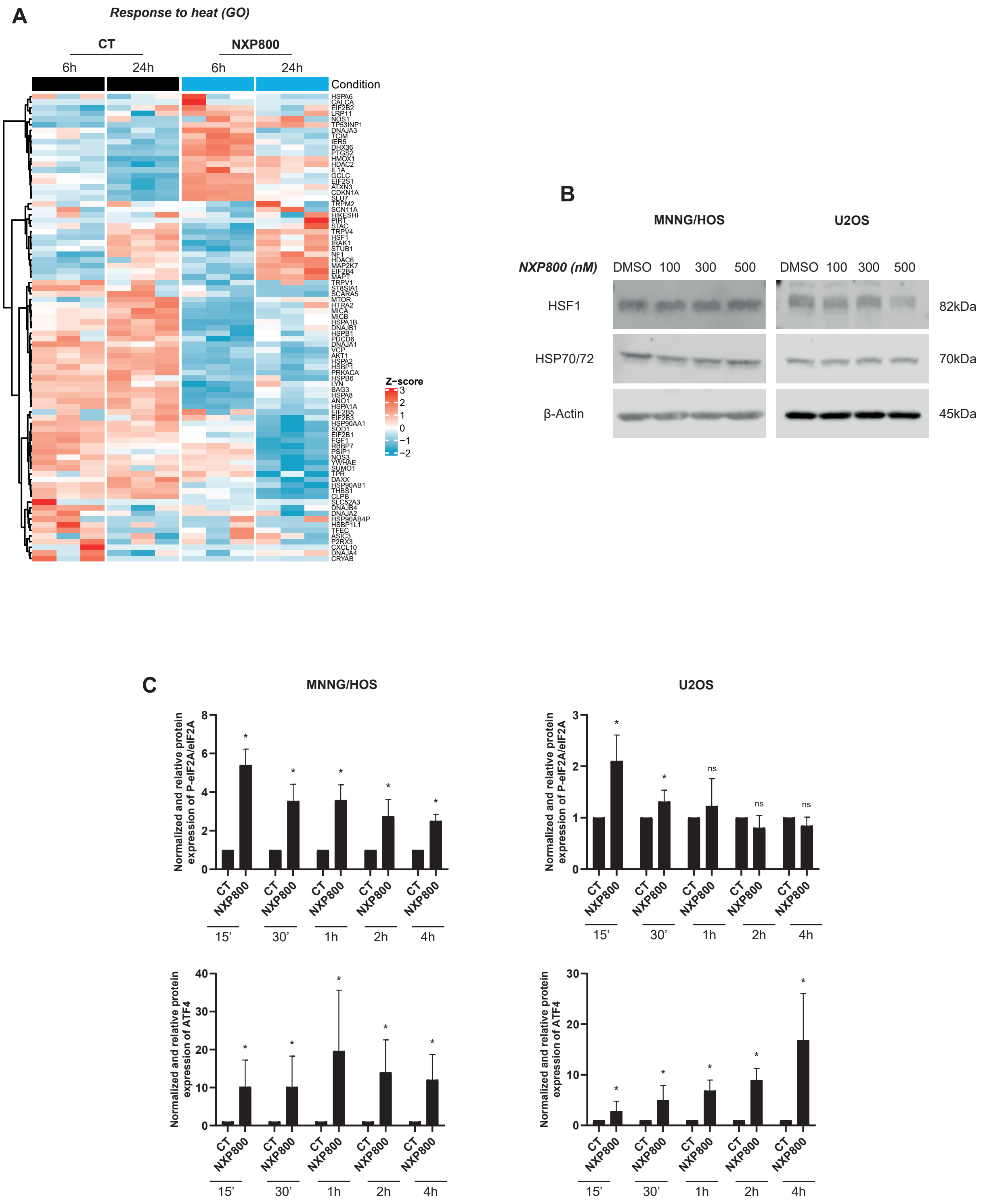

Figure S2: (A) RNA-seq was performed on MNNG/HOS cells treated with DMSO (CT) or 300nM NXP800 for 6 hours and 24 hours (N=3). The heatmap of differentially expressed genes associated with the Response to heat pathway (GO) was generated from RNA-seq data. (B) MNNG/HOS and U2OS cells were treated with indicated concentrations of NXP800 for 24 hours. The expression of HSF1 and HSP70/72 was assessed by Western blot (representative images of three independent biological replicates). (C) Quantification of Western blot (N=3, from figure 1F) was performed by densitometric analysis, normalized to β-Actin and expressed relative to CT. In C bars indicate means ± SD of the different values, *: $p < 0.05$ (Mann-Whitney test).

Figure S3

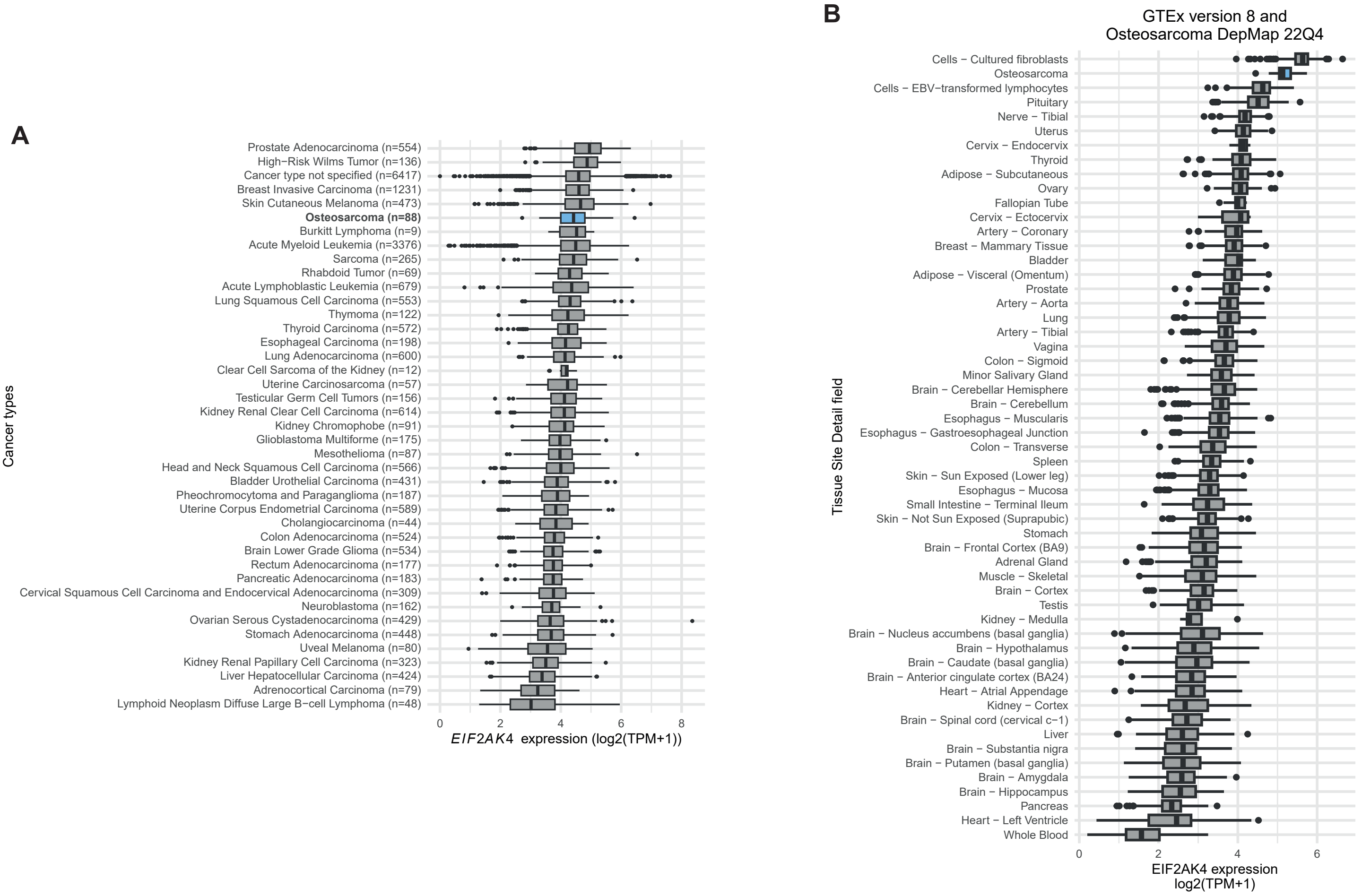

Figure S3: (A) EIF2AK4 expression in OS compared to tumor cell lines and (B) healthy tissues respectively based on CCLE and GTEx database.

Figure S4

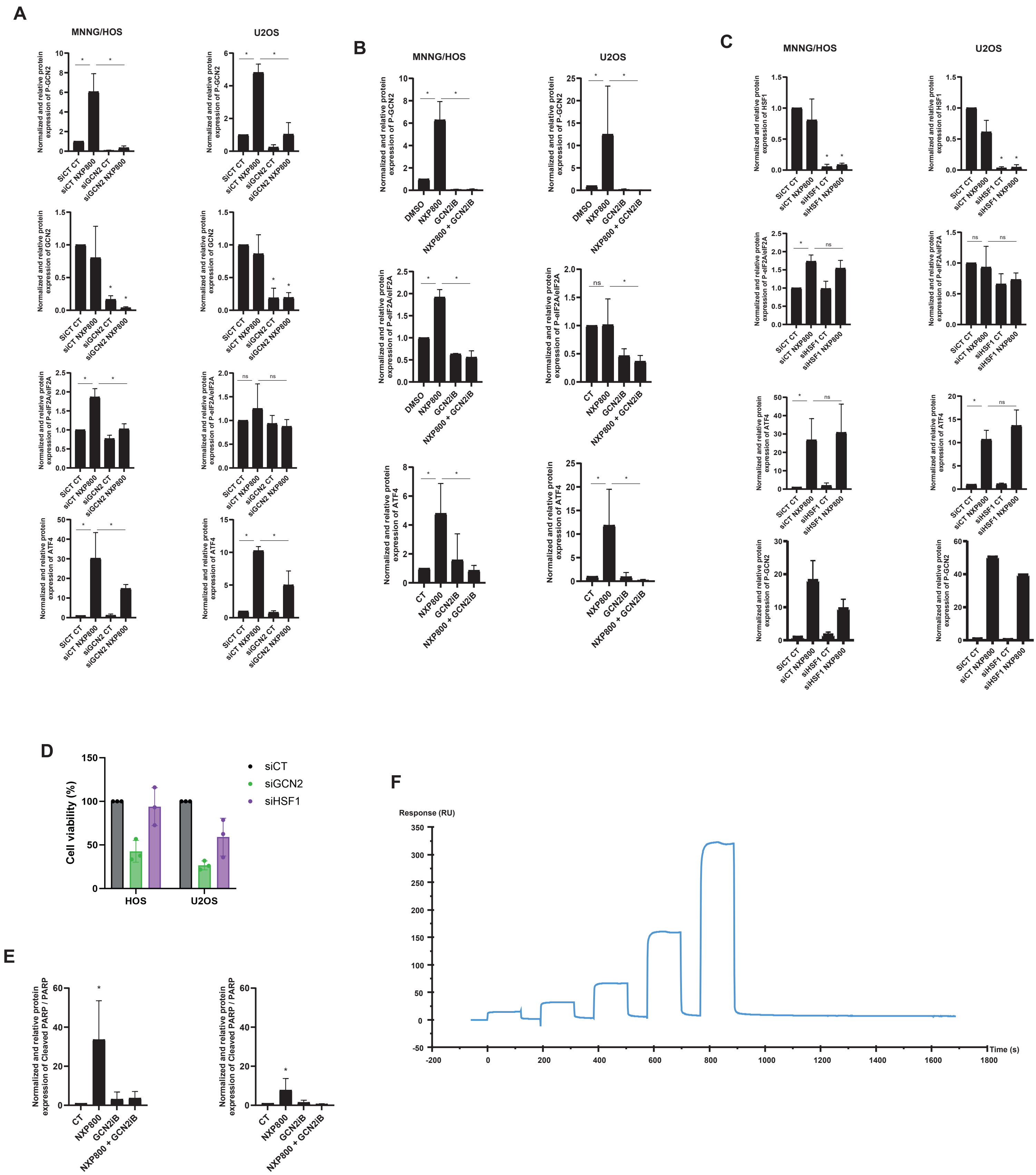

Figure S4: Quantification of Western blot from (A) figure 3E (N=3), (B) figure 3F (N=3), (C) figure 3G (N=3) was performed by densitometric analysis, normalized to β-Actin or Vinculin and expressed relative to control (untreated siCT or CT). (D) Cells were transfected for 24 hours with siCT, siHSF1, or siGCN2, re-seeded for 24 hours, and then treated with increasing concentrations of NXP800 (from 3.9nM to 1µM). Cell viability for each siRNA condition was assessed by crystal violet assay (N=3). (E) Quantification of Western blot (N=3, from figure 3J) was performed by densitometric analysis, normalized to β-Actin or Vinculin and expressed relative to CT. (F) Single-Cycle Kinetics sensorgrams of NXP800 (0–100 µM) injected over GCN2-GST (≈1500 RU) captured on an anti-GST functionalized CM5 biochip (association 120 seconds, dissociation 800 seconds). Bars indicate means ± SD of the different values, *: $p < 0.05$ (Mann-Whitney test).

Figure S5

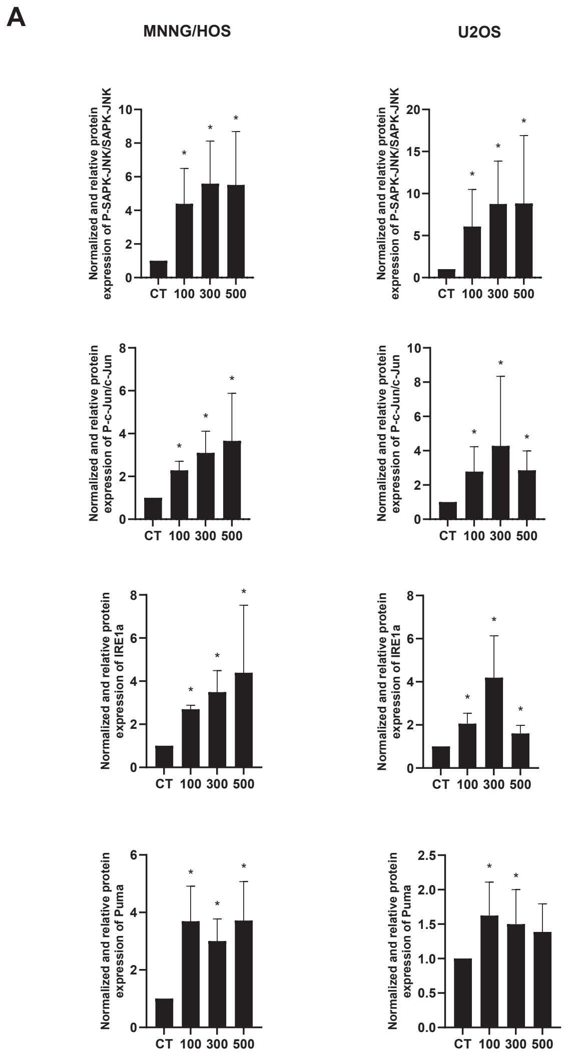

Figure S5: (A) Quantification of Western blot (N=3, from figure 4B) was performed by densitometric analysis, normalized to β-Actin and expressed relative to CT. Bars indicate means ± SD of the different values, *: $p < 0.05$ (Mann-Whitney test).

Figure S6

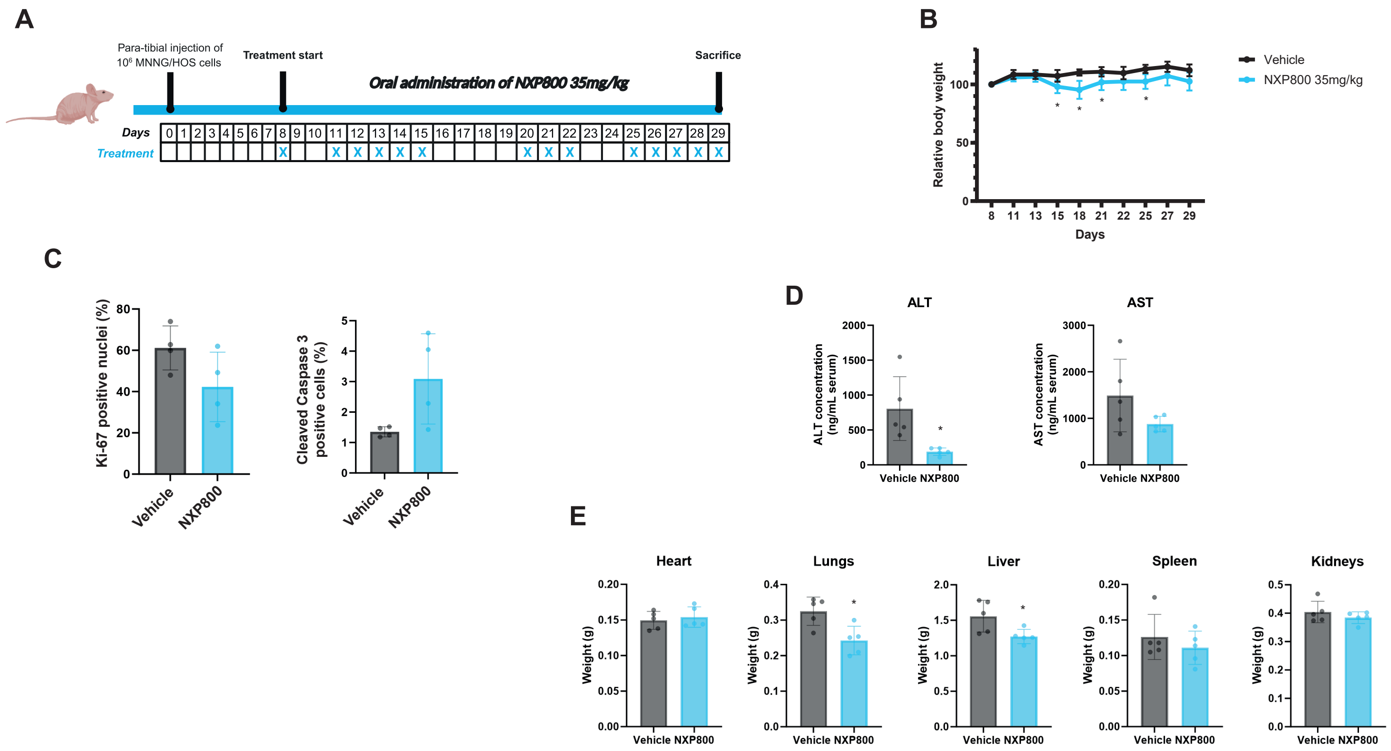

Figure S6: (A) Detailed protocol and therapeutic treatment regimen; (B) body weights were measured three times a week. (C) Immunostaining of Ki-67 and cleaved caspase -3 on tumor sections from mice grafted with 2.10^6 MNNG/HOS cells and treated daily for 5 days were performed and positive cells were quantified using QuPath software to assess cell proliferation and apoptotic activity. (D and E) Tumor-free NMRI nude mice were treated 5 days per week for 2 weeks. (D) Blood samples were collected by intracardiac puncture, and serum AST and ALT enzyme levels were quantified by ELISA. (E) The collected organs were weighed after sacrifice. In B, bars indicate means ± SEM, in C, D and E, bars indicate means ± SD of the different values; *: $p < 0.05$ (Mann Whitney test); ****: $p < 0.001$ (Anova test).
