# Supplementary material for: Activating GCN2 and subsequently the Unfolded Protein Response with the small oral molecule NXP800 delays tumor growth in osteosarcoma
Source: Cell Death Discov. 2026 Feb 5;12:94. doi: 10.1038/s41420-026-02941-2 (PMC12895007; doi:10.1038/s41420-026-02941-2)

Uncropped Western Blot

Details on Antibody Detection by Western Blot

Western blot analyses were performed as described in the Materials and Methods section. Primary antibodies were incubated and subsequently revealed in sequential steps. The numbers indicate the order in which the different antibodies were applied. Rabbit primary antibodies were detected using a secondary antibody conjugated to a fluorochrome with an emission wavelength of 700 nm, while mouse primary antibodies were detected using a secondary antibody conjugated to a fluorochrome with an emission wavelength of 800 nm. When multiple membranes are presented in the same figure, they are labeled with letters for clarity.

Fig 1E

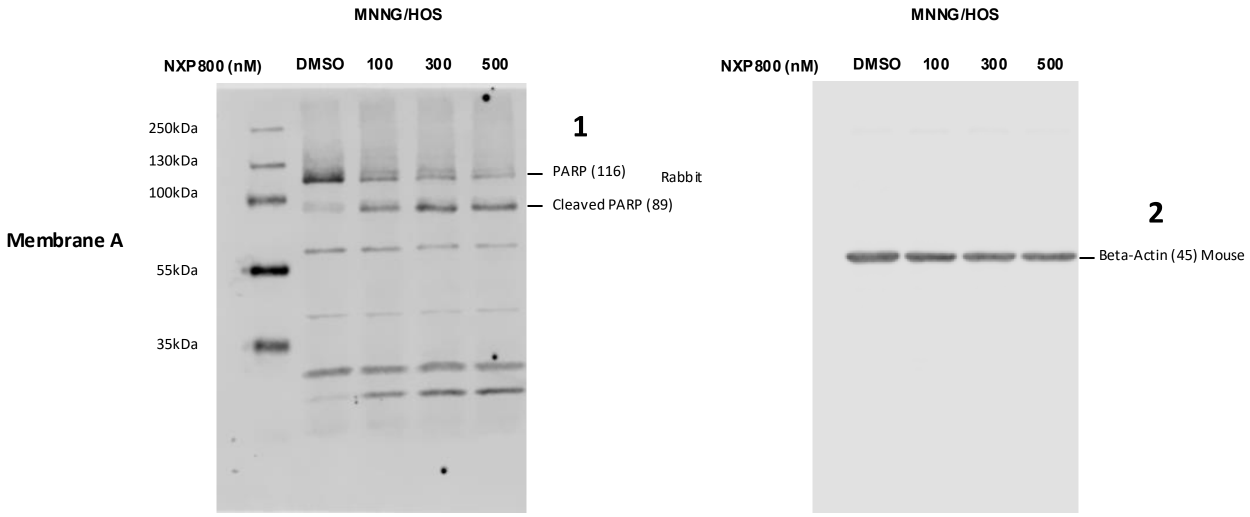

MNNG/HOS
NXP800 (nM)
DMSO
100
300
500
250kDa
130kDa
100kDa
55kDa
35kDa
1
PARP (116)
Rabbit
Cleaved PARP (89)
Membrane A
MNNG/HOS
NXP800 (nM)
DMSO
100
300
500
2
Beta-Actin (45) Mouse

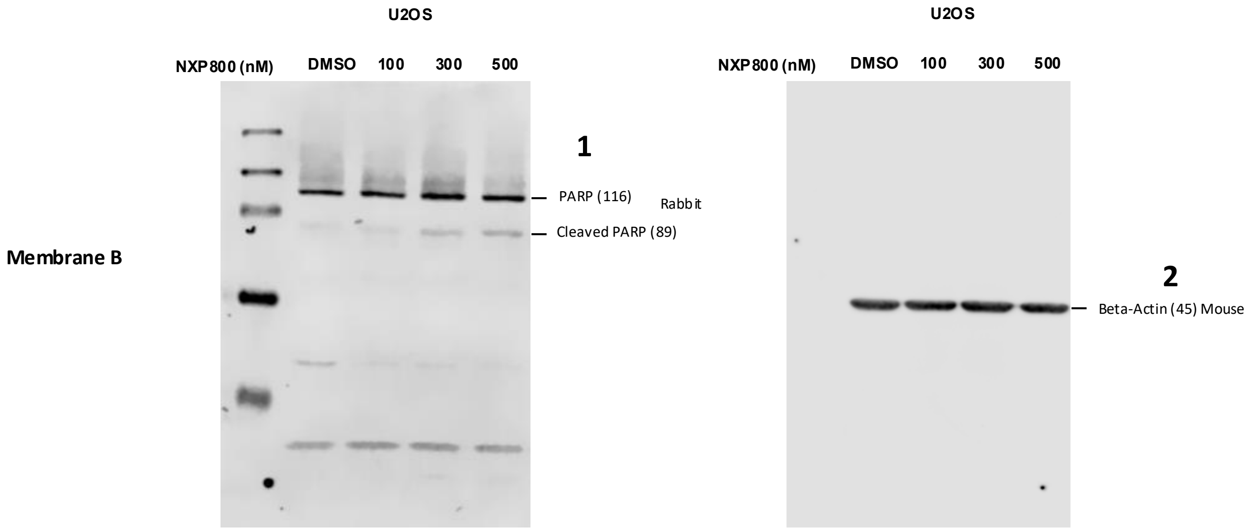

U2OS
NXP800 (nM)
DMSO
100
300
500
1
PARP (116)
Rabbit
Cleaved PARP (89)
Membrane B
U2OS
NXP800 (nM)
DMSO
100
300
500
2
Beta-Actin (45) Mouse

Fig 2F

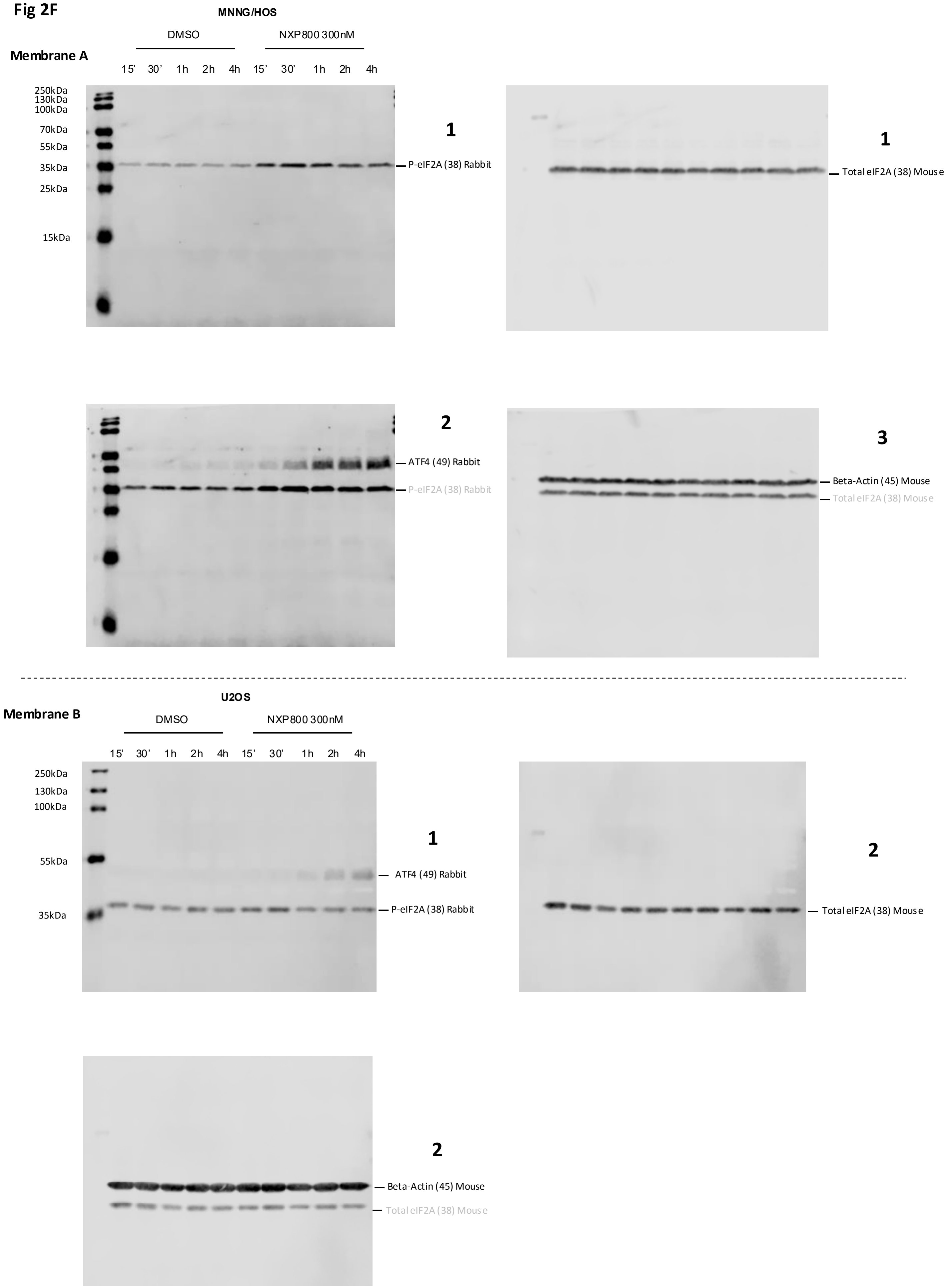
MNNG/HOS
DMSO
NXP800 300nM
Membrane A
15' 30' 1h 2h 4h 15' 30' 1h 2h 4h
250kDa
130kDa
100kDa
70kDa
55kDa
35kDa
25kDa
15kDa
1
P-eIF2A (38) Rabbit
1
Total eIF2A (38) Mouse
2
ATF4 (49) Rabbit
P-eIF2A (38) Rabbit
3
Beta-Actin (45) Mouse
Total eIF2A (38) Mouse
U2OS
Membrane B
DMSO
NXP800 300nM
15' 30' 1h 2h 4h 15' 30' 1h 2h 4h
250kDa
130kDa
100kDa
55kDa
35kDa
1
ATF4 (49) Rabbit
P-eIF2A (38) Rabbit
2
Total eIF2A (38) Mouse
2
Beta-Actin (45) Mouse
Total eIF2A (38) Mouse

Fig 3A

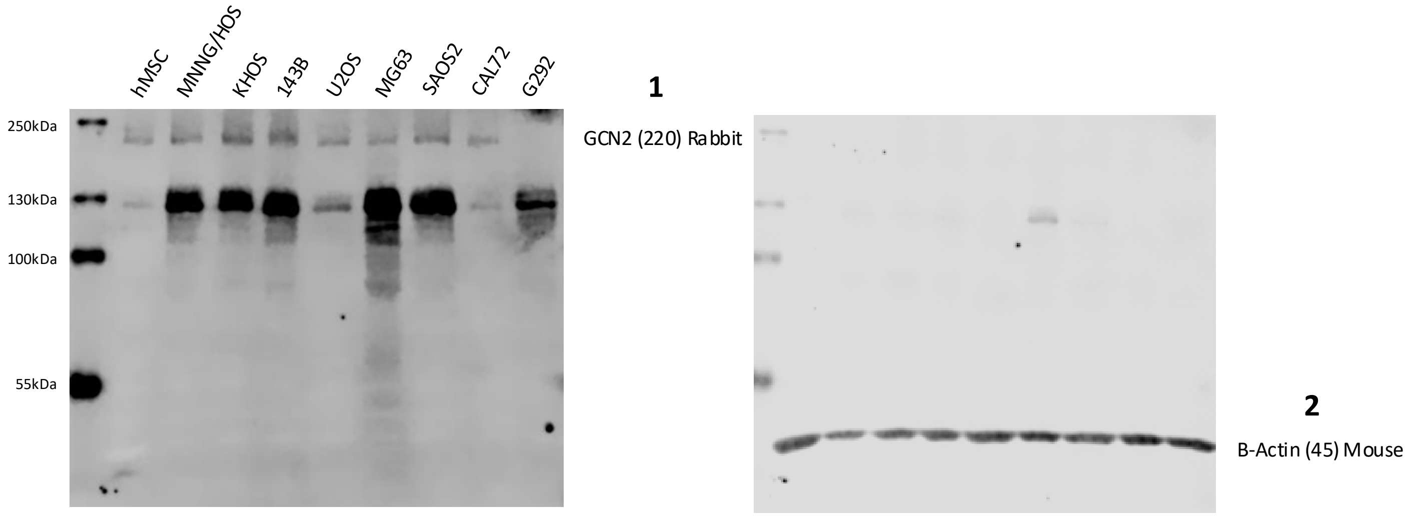
hMSC
MNNG/HOS
KHOS
143B
U2OS
MG63
SAOS2
CAL72
G292
250kDa
130kDa
100kDa
55kDa
1
GCN2 (220) Rabbit
2
B-Actin (45) Mouse

Fig 3E

MNNG/HOS siCT vs siGCN2

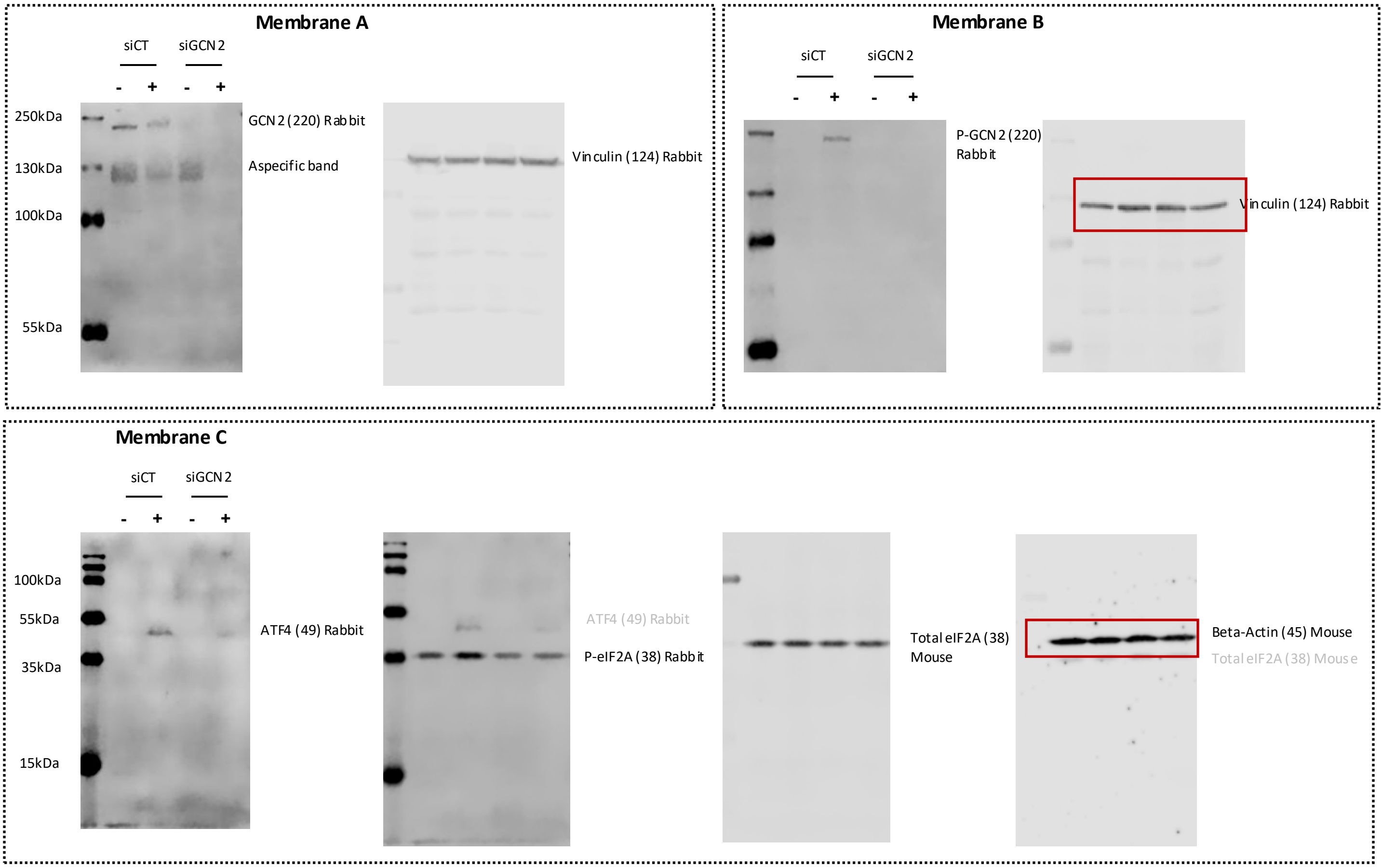

U2OS siCT vs siGCN2

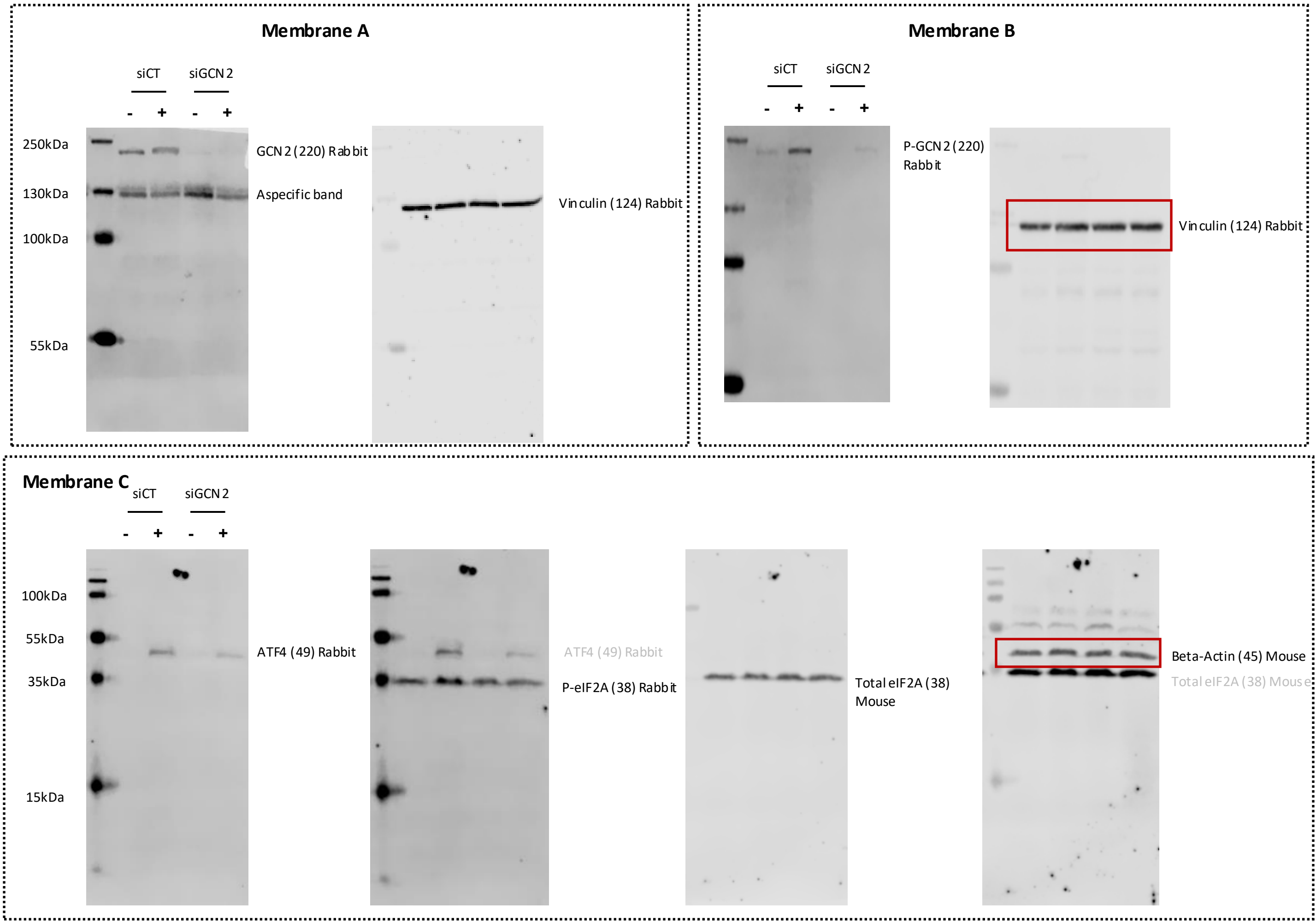

Fig 3F

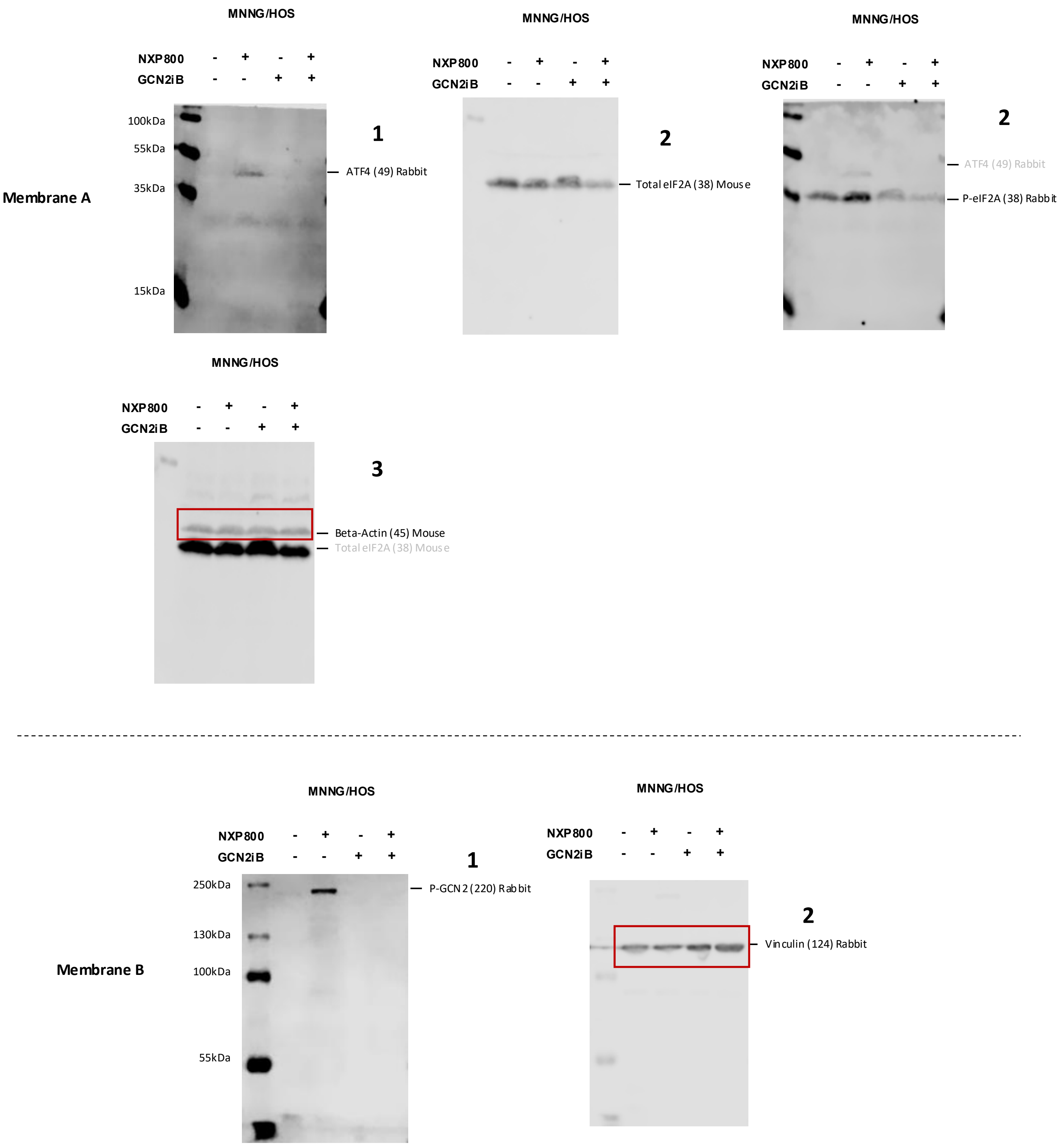
MNNG/HOS
NXP800 - + - +
GCN2iB - - + +
100kDa
55kDa
35kDa
15kDa
1
ATF4 (49) Rabbit
Membrane A
2
Total eIF2A (38) Mouse
ATF4 (49) Rabbit
P-eIF2A (38) Rabbit
3
Beta-Actin (45) Mouse
Total eIF2A (38) Mouse
Membrane B
250kDa
130kDa
100kDa
55kDa
P-GCN2 (220) Rabbit
Vinculin (124) Rabbit

Fig 3F

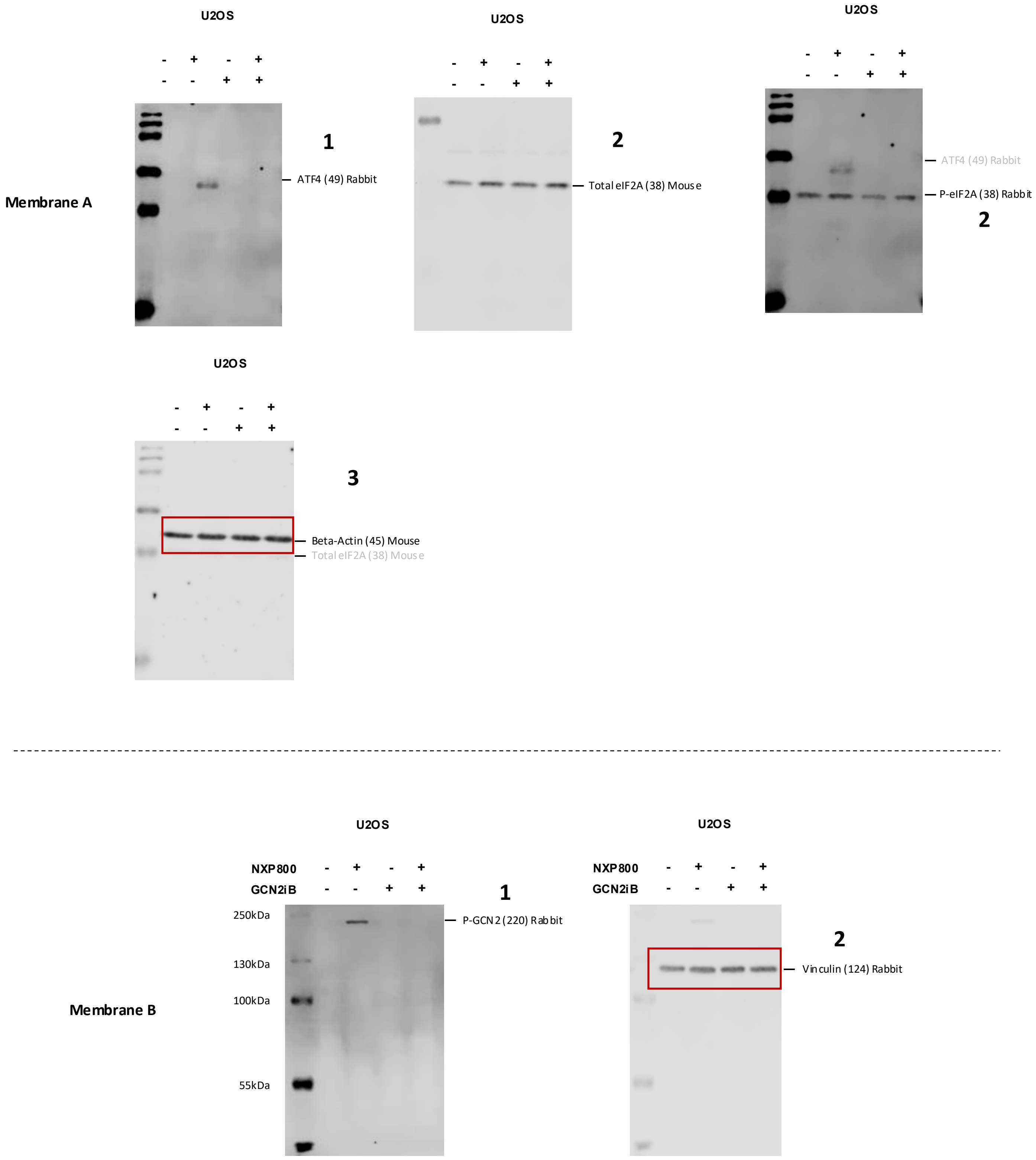
Membrane A
U2OS
1
ATF4 (49) Rabbit
2
Total eIF2A (38) Mouse
ATF4 (49) Rabbit
P-eIF2A (38) Rabbit
3
Beta-Actin (45) Mouse
Total eIF2A (38) Mouse
Membrane B
NXP800
GCN2iB
250kDa
130kDa
100kDa
55kDa
P-GCN2 (220) Rabbit
Vinculin (124) Rabbit

Fig 3G MNNG/HOS and U2OS siCT vs siHSF1

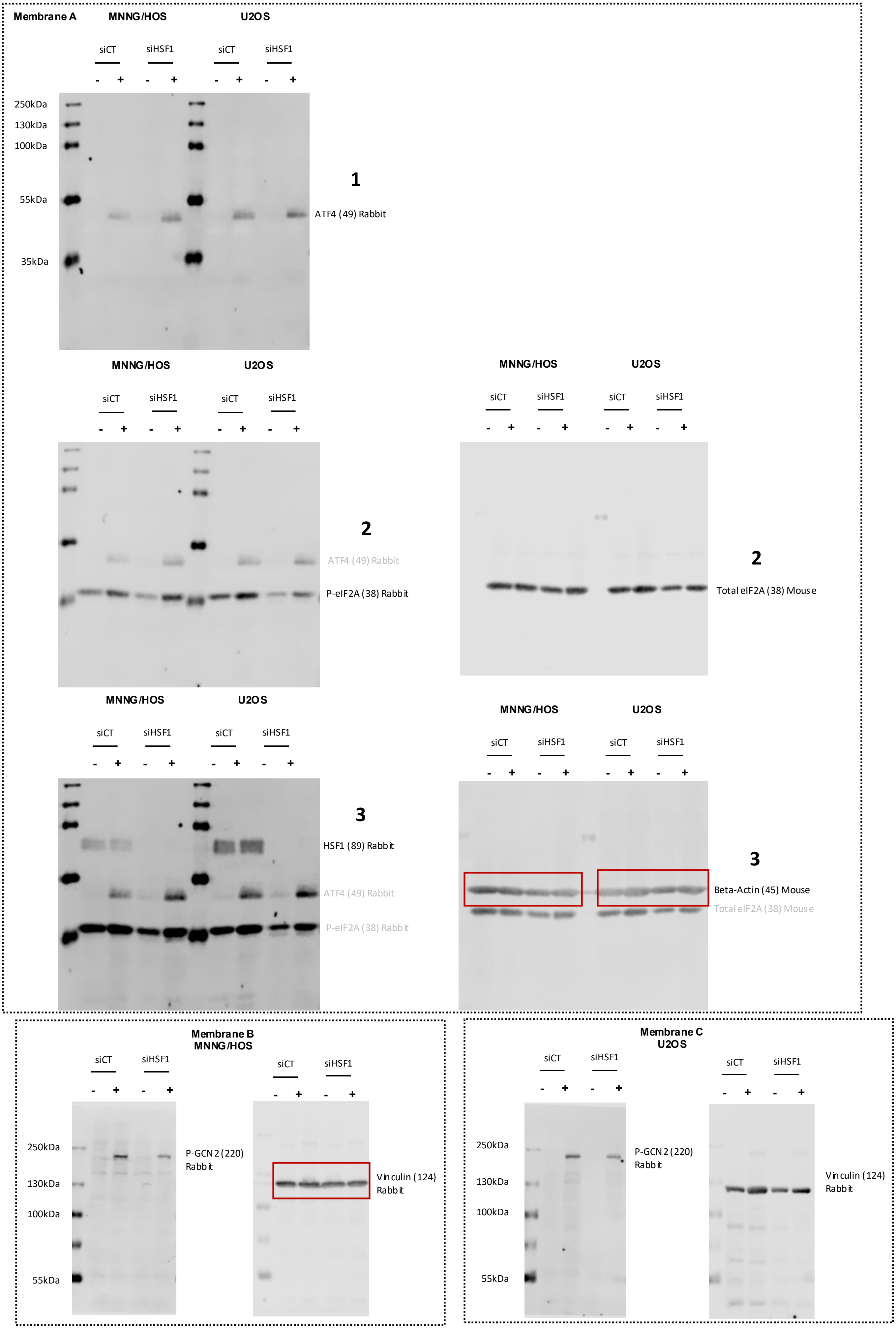

Fig 3J

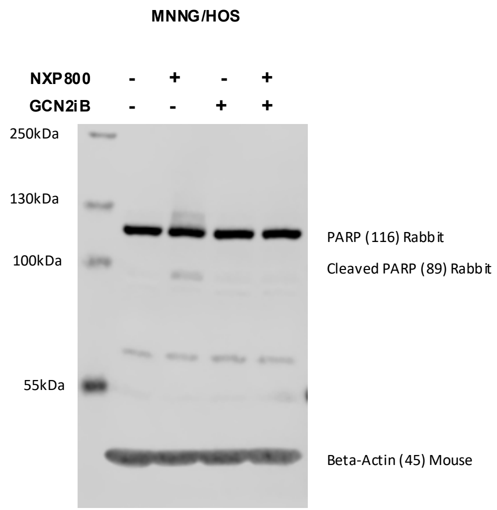
MNNG/HOS
NXP800 - + - +
GCN2iB - - + +
250kDa
130kDa
100kDa
55kDa
PARP (116) Rabbit
Cleaved PARP (89) Rabbit
Beta-Actin (45) Mouse

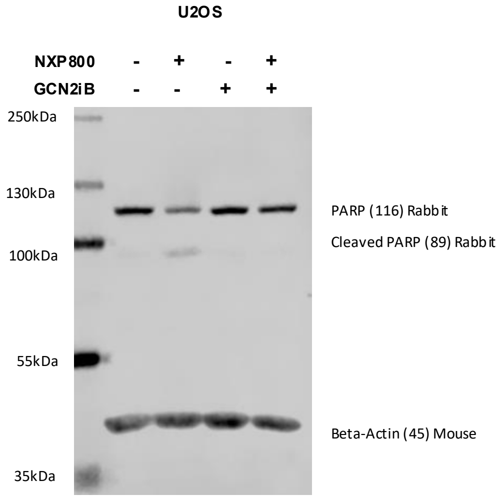
U2OS
NXP800 - + - +
GCN2iB - - + +
250kDa
130kDa
100kDa
55kDa
35kDa
PARP (116) Rabbit
Cleaved PARP (89) Rabbit
Beta-Actin (45) Mouse

Fig 4B – MNNG/HOS

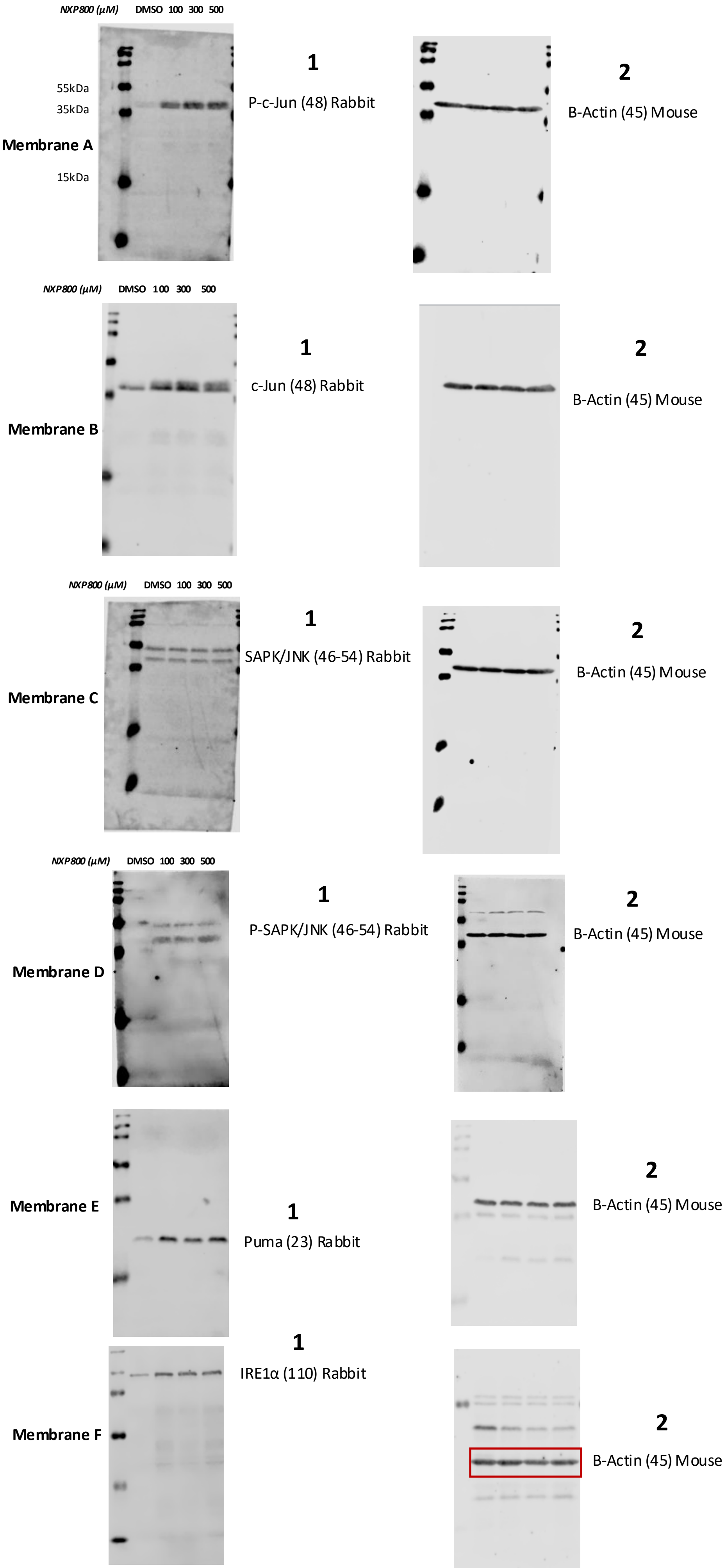

Fig 4C – U2OS

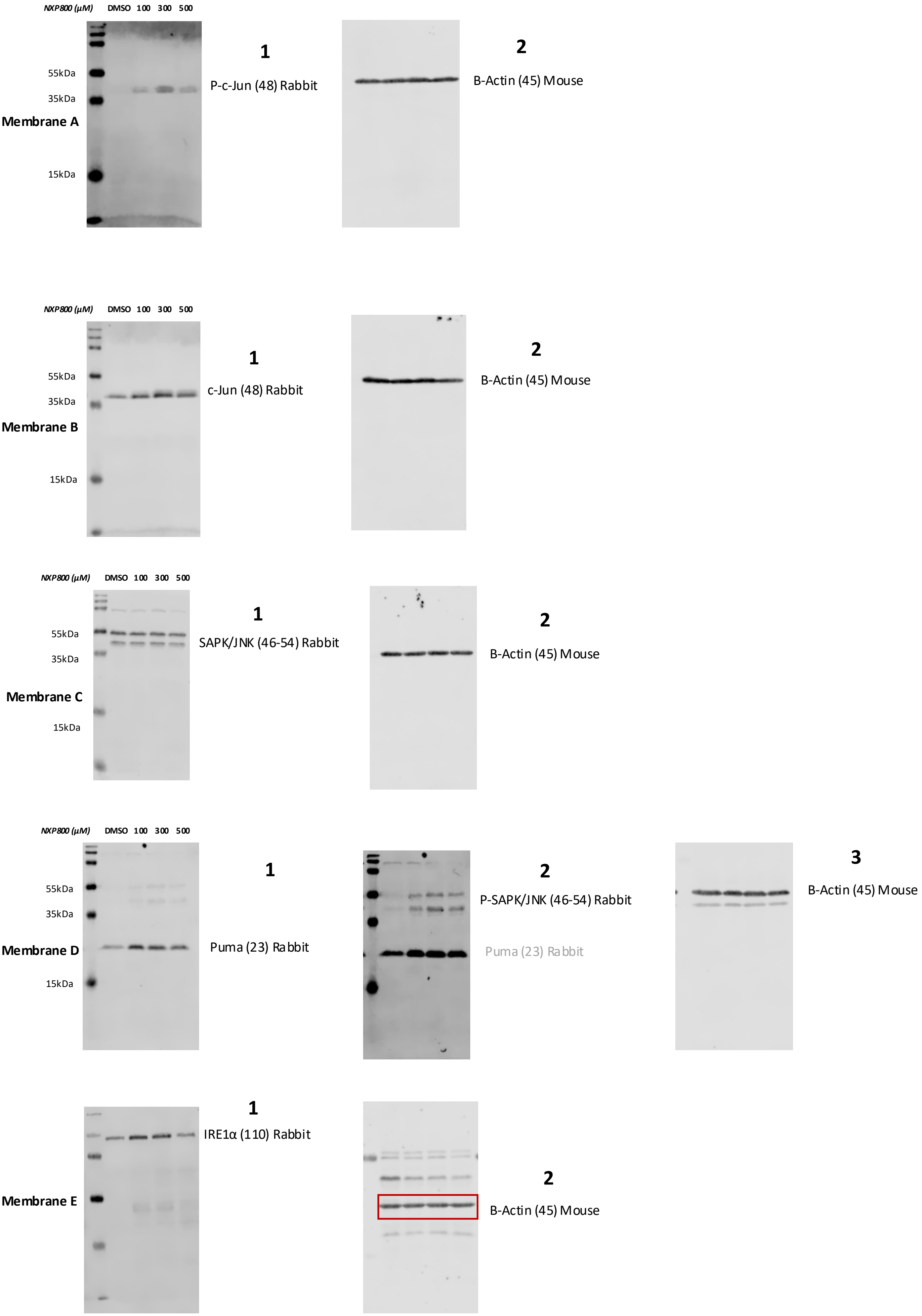
NXP800 (µM) DMSO 100 300 500
55kDa
35kDa
15kDa
Membrane A
1
P-c-Jun (48) Rabbit
2
B-Actin (45) Mouse
NXP800 (µM) DMSO 100 300 500
55kDa
35kDa
15kDa
Membrane B
1
c-Jun (48) Rabbit
2
B-Actin (45) Mouse
NXP800 (µM) DMSO 100 300 500
55kDa
35kDa
15kDa
Membrane C
1
SAPK/JNK (46-54) Rabbit
2
B-Actin (45) Mouse
NXP800 (µM) DMSO 100 300 500
55kDa
35kDa
15kDa
Membrane D
1
Puma (23) Rabbit
2
P-SAPK/JNK (46-54) Rabbit
Puma (23) Rabbit
3
B-Actin (45) Mouse
Membrane E
1
IRE1α (110) Rabbit
2
B-Actin (45) Mouse

Fig S1B

MNNG/HOS

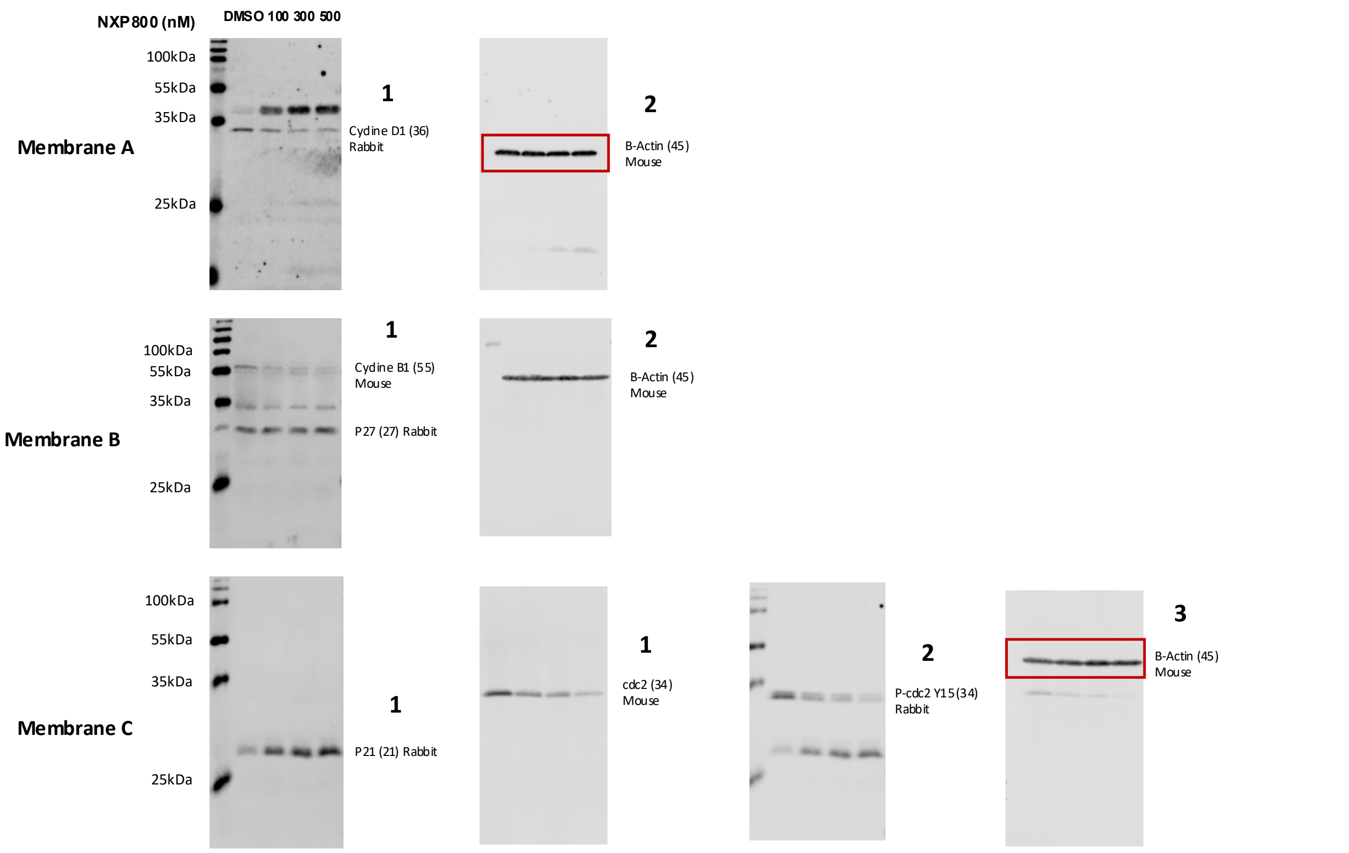

U2OS

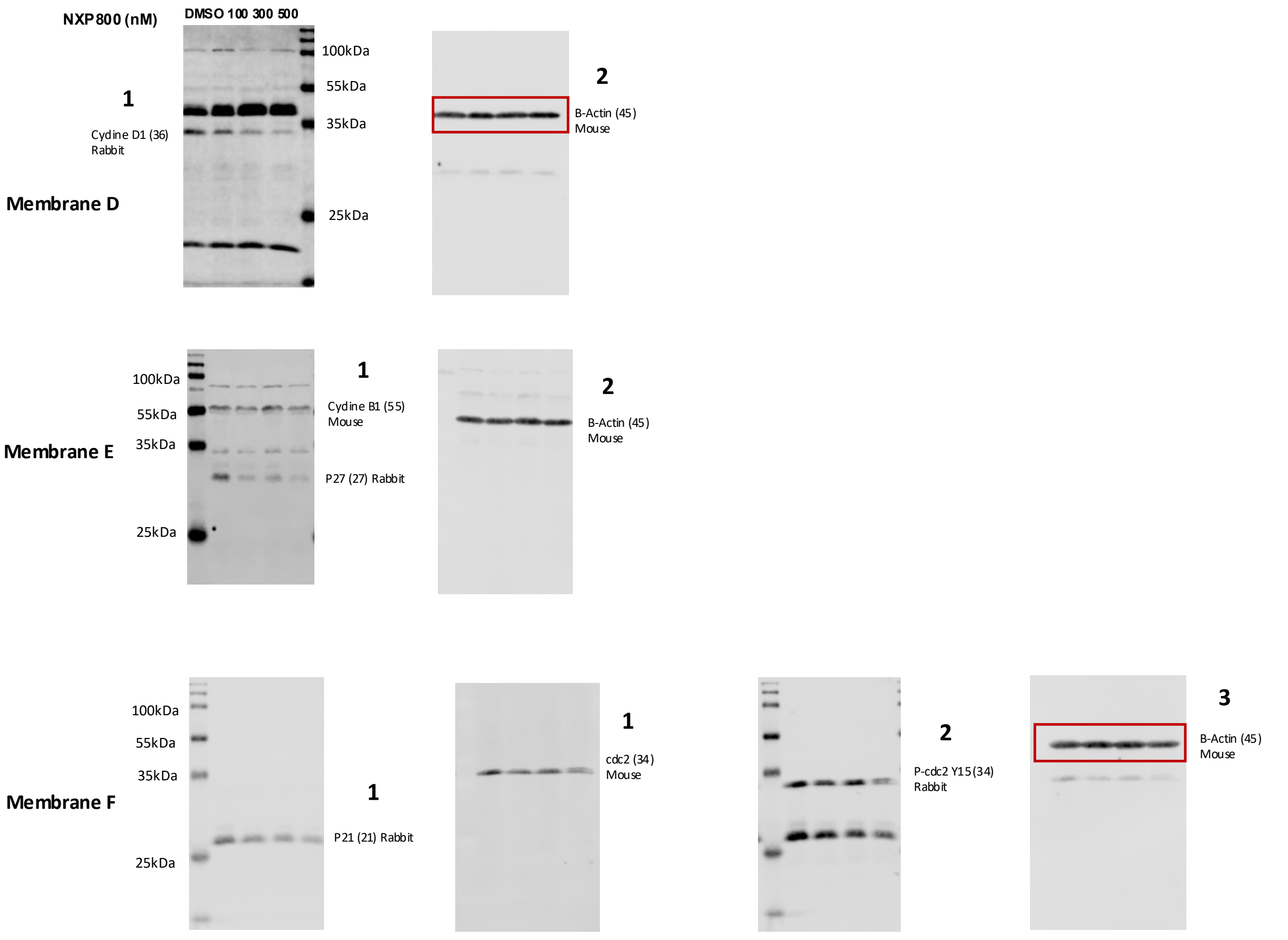

Fig S2B

MNNG/HOS

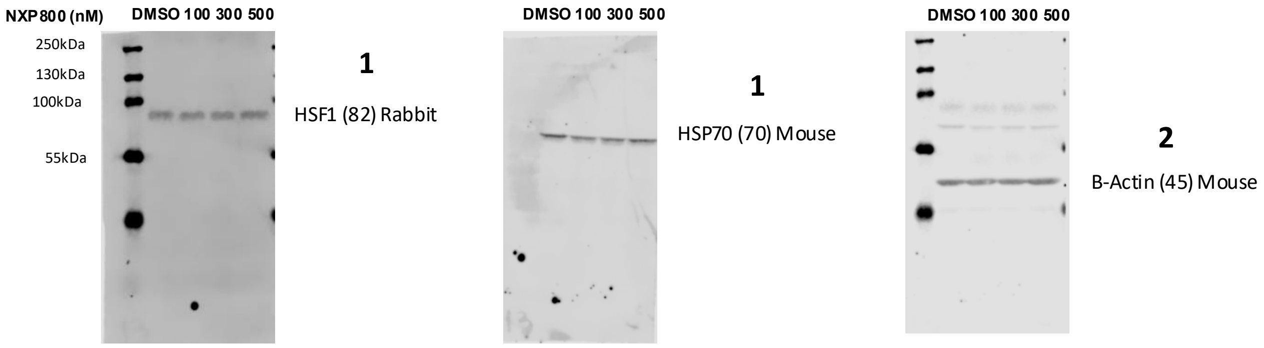

U2OS

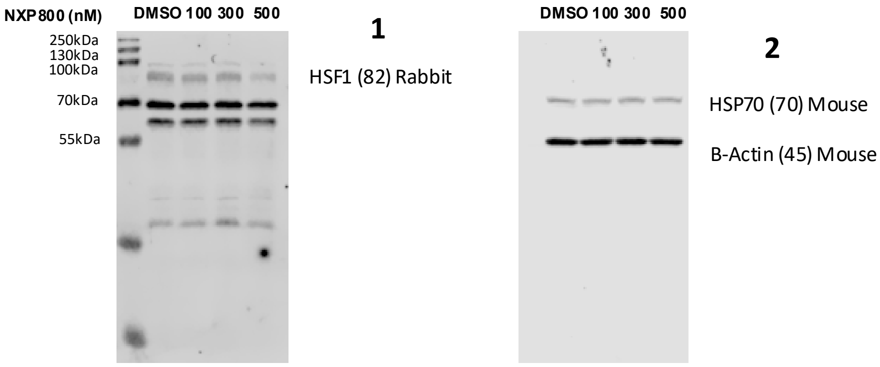

Supplement: Supplementary file 3 — Uncropped WB [file 41420_2026_2941_MOESM3_ESM.pdf]
